# An integrated approach utilizing raman spectroscopy and chemometrics for authentication and detection of adulteration of agarwood essential oils

**DOI:** 10.3389/fchem.2022.1036082

**Published:** 2022-12-21

**Authors:** Xiaoying Huang, Huiting Li, Yinlan Ruan, Zhen Li, Huda Yang, Guixin Xie, Yi Yang, Qing Du, Kaidi Ji, Ming Yang

**Affiliations:** ^1^ Key Laboratory of Modern Preparation of Traditional Chinese Medicine, Ministry of Education, Jiangxi University of Traditional Chinese Medicine, Nanchang, China; ^2^ School of Optoelectronic Engineering, Guilin University of Electronic Technology, Guilin, China; ^3^ Jiangxi Guxiangjinyun Great Health Industry Co. Ltd, Nanchang, China

**Keywords:** agarwood, adulteration, classification, *Aquilaria sinensis (Lour.) gilg*, volatile oil, raman spectroscopy, chemometrics

## Abstract

Agarwood is a precious aromatic plant which has good pharmacological effects such as antidepressant and sedation. It also has good ornamental and collection value. However, due to it is long and complex production process, the output of agarwood essential oils (AEOs) is scarce, so the price is expensive, the quality is uneven, and the adulteration events is endless. From the commercial and pharmaceutical point of view, the authenticity and quality of the commercial products labeled as AEOs is very important. This paper tested the applicability of Raman spectroscopy combined with chemometrics in classification and authenticity identification of AEOs. In this study, Raman spectroscopy and principal component analysis (PCA) combined with partial least square discriminant analysis (PLS-DA) were used to comprehensively evaluate AEOs from different geographical origins and/or extracted by different methods which showed different characteristic bands. The characteristic component of AEOs, chromone derivatives, and two commonly used adulterants were also detected. These characteristic bands provide spectrum information of AEO samples and reference materials, which can be used as Raman spectral markers for the qualitative identification of AEOs. This study can provide a novel, fast and convenient method for identification of AEOs.

## 1 Introduction

Agarwood is a precious aromatic plant and has been used as a traditional medicine in China and Southeast Asian countries. Volatile oils are the main effective substances of the agarwood, and their characteristic components include sesquiterpenes, 2-(2-phenylethyl) chromone derivatives and agarofurans. Studies have proved that agarwood essential oils (AEOs) have good pharmacological effects such as antidepressant, sedative and anti-inflammatory (Hashim et al., 2016). AEOs is not only a medicine with rich pharmacological activity, it is also widely used in food, perfume, collection, religion and other fields. As a commodity with high commercial value, the wholesale price of high-quality AEOs is about US$30,000–US$50,000 per liter which led to adulteration of the AEOs on the market (Persoon and Beek, 2008; Hidayat et al., 2010). In addition, the compositions of the AEOs are affected by many factors such as geographical origins, extraction methods, botanic origins and so on, which may have large impact on its medical functions. The AEOs are mainly produced in Southeast Asian countries, such as Guangdong Province (China), Hainan Province (China), Malaysia, Indonesia, Vietnam and so on. The source of Chinese agarwood is *Aquilaria sinensis (Lour.) gilg*, a plant of Thymelaeaceae in *Daphne* family, which contains resin. The agarwood produced in Vietnam, Malaysia and other Southeast Asian countries mainly come from *A. malaccensis Lamk.*, *A. crasna*, *etc.* of *Aquilaria.* At present, the most common extraction methods of AEOs are steam distillation extraction (SDE) and supercritical fluid extraction (SFE). Due to the high value of AEOs, their adulteration is very common. Merchants will deceive consumers by adding plasticizers such as diethyl phthalate and diethylhexyl phthalate (DEHP) in AEOs, so as to seek higher interests (Yang et al., 2016). Therefore it is highly necessary to analyze the quality of the AEOs to reveal their origins and authenticity.

At present, GC-MS and LC-MS are the most used methods in the quality evaluation of AEOs, they require complex sample preparation, thus are time-consuming with high analysis cost (Rodríguez-Solana et al., 2014). It is thus necessary to develop more rapid methods to effectively achieve quality control and classification. As a fingerprint analysis technology, Raman spectroscopy has great potential to meet these requirements. Raman spectrum is a vibrational spectrum, which can obtain information such as molecular structure, conformation, intermolecular interaction and chemical bond (Velioglu et al., 2017). It can be used for rapid detection and quality evaluation of volatile oil samples, qualitative and quantitative analysis. Compared with other analysis methods, it has unique advantages in the analysis of essential oil samples, such as nondestructive, less analysis dosage, and fast and convenient detection process (Rodríguez-Solana et al., 2014).

**FIGURE 1:**
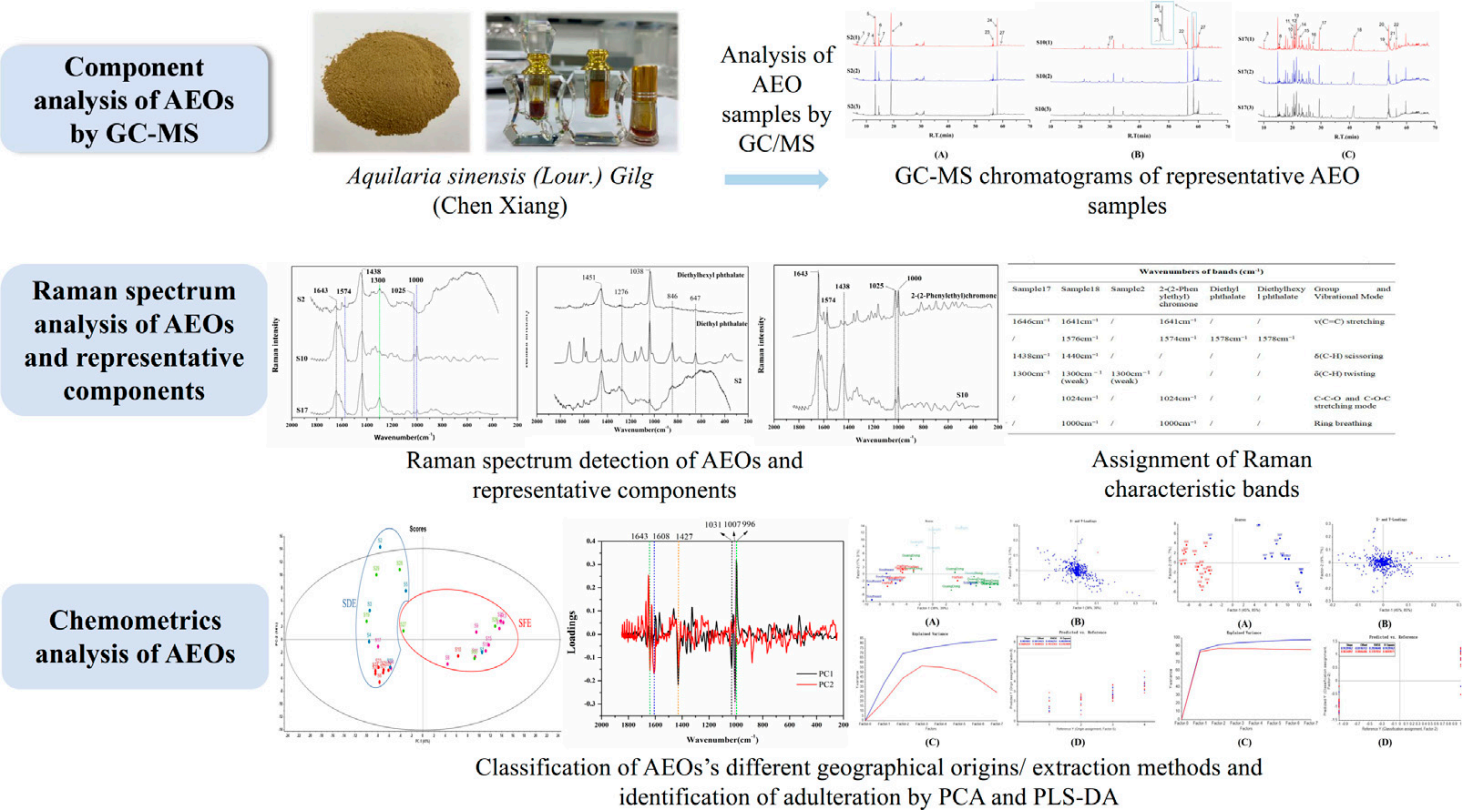
A schematic diagram of the research.

In this study, Raman spectroscopy was used to evaluate 30 batches of commercial AEOs from different geographical origins and made by different extraction methods. The observed Raman bands were assigned, and the spectral data were analyzed by chemometrics to identify adulterated samples, evaluate the quality of different AEO samples, and determine their Raman characteristic bands as the basis for AEOs classification and identification. Subsequently, two chemometrics methods, PCA and PLS-DA, were combined for AEOs’ authentication. As a dimension reduction analysis method, Principal component analysis (PCA) is widely used to deal with multivariable problems in the field of spectral data analysis (Lee et al., 2018). It can reduce the data dimension by obtaining a small number of principal component variables to explain the complex original data information. It can not only ensure the integrity of the original data, but also reduce the dimension of high-dimensional variables, so as to accurately, objectively and comprehensively evaluate and distinguish the spectra between different products and classify multiple samples (Hu et al., 2019). However, PCA is an unsupervised pattern recognition method, and the accuracy of the analysis results needs to be strengthened. Therefore, the supervised pattern recognition method of Partial least square discriminant analysis (PLS-DA) was used to further evaluate the Raman data of AEOs. Through PLS-DA analysis, variable data and classification information of collected data set were divided into calibration and a validation data subset (Velioglu et al., 2017). The dimension reduction analysis was combined with different categories of AEOs to highlight the differences between groups, so as to further distinguish each type of AEO samples. In this research, Raman spectroscopy combined with PCA and PLS-DA were used to develop a novel, convenient and effective method for rapid determination of AEOs. The technical strategy of this study is shown in Figure 1.

## 2 Materials and methods

### 2.1 Materials and reagents

Anhydrous sodium sulfate (AR, Xilong Technology Co., Ltd.) and anhydrous ethanol (AR, Xilong Technology Co., Ltd.) were used. Standards of 2-(2-Phenylethyl) chromone (20 mg, purity 98%), 6,7-dimethoxy-2-(2-phenylethyl) chromone (10 mg, purity 98%), benzylacetone (25 ml, purity 98%), palmitic acid (20 mg, purity 98%), diethyl phthalate (1 ml, purity 98%) and diethylhexyl phthalate (20 mg, purity 98%) were purchased from Chengdu Alfa Biotechnology Co. Ltd.

### 2.2 Sample preparation of AEOs

30 batches of AEO samples were extracted in two methods (SDE and SFE), which came from seven geographical areas including Hainan Province (China), Guangdong Province (China), Guangxi Province (China), Cambodia, Indonesia, Laos and Thailand. The detailed information of geographical origins and extraction methods are shown in Table 1. They were purchased from local businesses in China. For this work, the samples were labeled S1 to S30 and stored away from light at room temperature.

**TABLE 1:** Geographical origins and extraction methods of 30 batches of AEO samples.

No.	Origin	Extraction method	No.	Origin	Extraction method
S1	Cambodia	SFE	S16	Guangdong, China	SDE
S2	Indonesia	SDE	S17	Guangdong, China	SDE
S3	Indonesia	SDE	S18	Guangdong, China	SFE
S4	Laos	SDE	S19	Guangxi, China	SDE
S5	Thailand	SDE	S20	Hainan, China	SDE
S6	Hainan, China	SDE	S21	Hainan, China	SDE
S7	Hainan, China	SDE	S22	Laos	SDE
S8	Guangdong, China	SFE	S23	Hainan, China	SDE
S9	Guangdong, China	SFE	S24	Hainan, China	SDE
S10	Hainan, China	SFE	S25	Guangdong, China	SFE
S11	Guangdong, China	SFE	S26	Guangxi, China	SFE
S12	Guangdong, China	SFE	S27	Guangxi, China	SFE
S13	Guangdong, China	SFE	S28	Guangxi, China	SDE
S14	Hainan, China	SDE	S29	Guangxi, China	SDE
S15	Guangdong, China	SFE	S30	Guangxi, China	SFE

### 2.3 Instruments

Raman Analyzer from Zhuhai Subphotonics Detection Co. Ltd. was used to measure the samples. It uses a 1064 nm laser with 500 mw maximum power to reduce background fluorescence of the AEOs and its wavenumber range is 200-2200 cm^−1^ with a spectral resolution of 10 cm^−1^. 7890a/5975c gas chromatography-mass spectrometer (Agilent Technology Co., Ltd.), and hp-5 ms capillary column (30 × 0.25 × 0.25 m) were used to analyze the samples for comparison.

### 2.4 Raman spectroscopy measurements

Raman spectra of 30 AEO samples were collected through a cuvette filled with 600 μl of each sample. The signal acquisition time was 15 s with 10 averaging.

### 2.5 Data pre-processing

The classification of the samples based on their Raman spectra were analyzed by using the Unscramabler 10.4 software. Several common techniques such as standard normal variable correction (SNV), baseline correction, data smoothing, derivative method (first and second order derivation) were used to pretreat Raman spectra. Before chemometric analysis, a preprocessing method of obtain the derivative first, and then do SNV standardization processing was used to preprocess the data (Omar et al., 2012).

### 2.6 Chemometric analysis

#### 2.6.1 Unsupervised pattern anlysis method of PCA

SIMCA-P software was used for PCA analysis in the region of 1850–246 cm^−1^ for the AEO samples to analyze the distribution of samples in the whole response surface. Two new matrices, scores and loadings, were used to represent the linear combination of the original variables. In the response surface, the tighter the two samples fit, the smaller the difference between individuals. On the contrary, the farther the difference is, the larger the individual difference is. By analyzing the position of the data on the response surface, circle a certain area to achieve the purpose of classification between samples.

#### 2.6.2 Supervised pattern anlysis method of PLS-DA

The Unscramabler 10.4 software was used for PLS-DA analysis. The collected 30 AEO samples were randomly divided into a calibration and a validation data subset, and PLS-DA model was established and validated. The spectral data of the calibration data subset was used as the input variable x, and the classification of samples was assigned in different extraction methods and different origins and used as the output variable y. The established model was tested with the Raman data of AEOs in the validation data subset as the unknown sample. The spectral data was imported into the PLS-DA model as an input variable, and the predictive value was calculated and compared with the assigned value, so as to distinguish the origin and extraction method of unknown samples. Finally, the accuracy of the discriminant model was evaluated according to the accuracy of the discriminant and the discrete degree of the predictive values.

### 2.7 GC-MS analysis

GC-MS was used to check the quality of the samples. The gas chromatographic conditions were as follows: Agilent HP-5MS (30 × 250 μm x 0.25 µm) capillary column was used, the carrier gas was high purity He (99.999%), the sample volume was l μl, the shunt ratio was 10:1, and the flow rate was 1 ml/min. The temperature rising program was controlled as follows: an initial temperature of 60 C (held for 2 min), increased by 10 C/min up to 140 C, increased by 1 C/min up to 180 C (held for 2 min), and then increased by 20 C/min up to 300 C (held for 10 min). The mass spectrometry conditions were as follows: an EI ion source, the electron energy was 70 eV, the ion source temperature was 230 C, the MS quadrupole temperature was 150 C, the interface temperature was 250 C, the solvent delay was 3.0 min, the quality scan pattern was full scan, and the scan range was 30–650 amu. The NIST 17.0 mass spectrum database, standards of characteristic components and C7-C40 n-alkanes were used to comprehensively identify the components. The retention index (relative to C7-C40 n-alkanes, under the same gas chromatographic conditions) of each compound was calculated and compared with literatures’ values.

## 3 Results

### 3.1 Results of GC-MS

**FIGURE 2:**
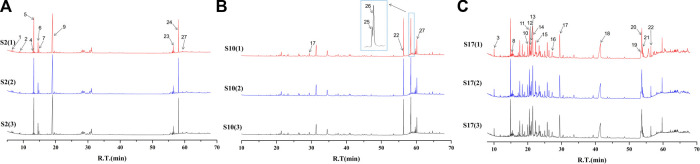
GC-MS chromatograms of representative AEO samples: **(A)** S2 (adulterated sample), **(B)** S10 (extracted by SFE), **(C)** S17 (extracted by SDE). (1)–(3) represents the GC-MS chromatograms of the same AEO sample, and three times GC-MS analysis were carried out to show the repeatability. The 26 components identified are numbered in the figure, which matches the serial numbers of each component in Table 2.

**FIGURE 3:**
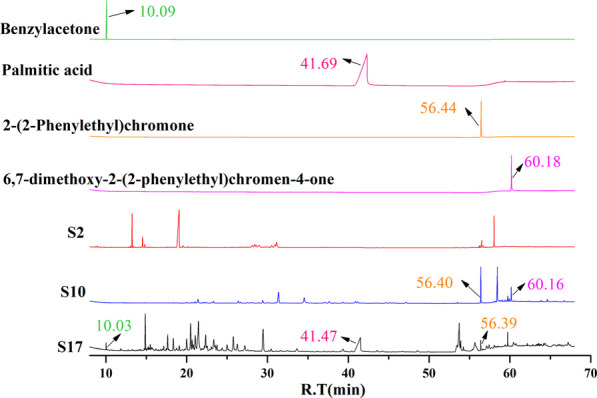
GC-MS chromatograms of representative AEO samples and comparison with four characteristic component standards.

The batches of AEOs were firstly analyzed by GC-MS, and the results showed that different extraction methods had a great influence on the compositions of AEO samples. Among the 30 AEO samples, S17 and S10 are representative samples of SDF and SFE extraction methods, respectively (Figures 2B,C). As shown in the chromatograms (Figure 2C), the 17 AEO samples extracted by SDE mainly contain sesquiterpenoids and aromatic compounds. Other components mainly include agarospirol (19.63 min) and dehydrofukinone (27.93 min), and a few SDE samples contain 2- (2-phenylethyl) chromone derivatives (S19, S20, S21, S22). GC-MS chromatograms of representative AEO samples and comparison with four characteristic component standards were shown in Figure 3.

**TABLE 2:** The components of three representative AEO samples.

Peak no.	Identification	R_T_ (min)	RIa	RIb	Relative percentage content of components in the three representative samples (%)		
					S2	S10	S17
1	Dipropylene Glycol	6.4618	997	1,030	0.4261	0	0
2	DL-Menthol	8.9474	1,149	1,174	0.274	0	0
3	Benzylacetone	10.0329	1,217	1,252	0	0	0.6022
4	Cyperene	13.0366	1,378	1,396	0.5639	0	0
5	(-)-α-gurjunene	13.2467	1,388	1,406	12.2664	0	0
6	(−)-Alloaromadendrene	14.542	1,437	1,467	4.3142	0	0
7	(+)-γ-Gurjunene	14.8221	1,447	1,473	1.5659	0	0
8	2,10,10-Trimethyltricyclo [7.1.1.0 (2,7)]undec-6-en-8-one	15.4944	1,470	—	0	0	1.0716
9	Diethyl phthalate	19.0653	1,570	1,597.82	45.3589	0	0
10	(+)-γ-Eudesmol	20.0037	1,595	1,629	0	0	2.8209
11	γ-Eudesmol	20.4938	1,606	1,635.65	0	0	5.9918
12	Agarospirol	20.6688	1,609	1,639.53	0	0	2.2136
13	8,8,9,9-Tetramethyl-3,4,5,6,7,8-hexahydro-2H-2,4a-methanonaphthalene	21.0609	1,617	1,679	0	0	4.1261
14	Alloaromadendrene	21.4531	1,625	1,649.01	0	0	12.2096
15	2,3,4,5-Tetramethyltricyclo [3.2.1.02,7] oct-3-ene	22.3493	1,643	—	0	0	3.0516
16	Tetradecanoic acid	27.2017	1732	1764	0	0	1.9146
17	Dehydrofukinone	29.4563	1769	1774.3	0	4.1162	6.9797
18	Palmitic acid	41.4717	1940	1962.97	0	0	13.8876
19	10(E),12(Z)-Conjugated linoleic acid	53.4591	2,104	2,129	0	0	2.7563
20	Oleic acid	53.7251	2,116	2,137.02	0	0	9.5565
21	1-Penten-3-one,1,5-diphenyl	53.9072	2,125	—	0	0	1.7956
22	2-(2-Phenylethyl)chromone	56.3929	2,538	2,613	0	17.9741	0.8581
23	Methyl dehydroabietate	56.5048	2,546	2,477	2.4296	0	0
24	Diethylhexyl phthalate	58.0383	2,640	2,540.09	5.8284	0	0
25	6-Methoxy-2-(2-phenylethyl) chromen-4-one	58.3955	2,824	—	0	6.2816	0
26	2-[2-(4-Methoxyphenyl)ethyl] chromen-4-one	58.4375	2,826	—	0	16.5532	0
27	6,7-Dimethoxy-2-(2-phenylethyl)chromen-4-one	60.16	3,004	2,959.2	3.2934	8.8895	0

RI, retention indices; R_T_, retention time.

RI^a^, calculated from R_T_ s, in relation to those of a series C_7_-C_40_ of n-alkanes.

RI^b^, from the literatures (Jerković et al., 2011; Su et al., 2012; Chen et al., 2017; Han et al., 2019; Heidary Jamebozorgi et al., 2019; Ben Hassine et al., 2021; Camele et al., 2021; Geng, 2021; Li et al., 2021; Abu-Darwish et al., 2022; Chen et al., 2022; Costa et al., 2022; Jaradat et al., 2022; Yao et al., 2022).

Based on the analysis of chromatographic results, it is speculated that the differences in the components of 30 AEO samples may be related to the differences in the process principles of the two extraction methods. SFE mainly uses some supercritical fluids, which have the dual properties of gas and liquid when the temperature and pressure are above the critical point, to penetrate deeper into the aromatic herbs, so as to extract volatile components with high boiling point and high molecular weight (Yousefi et al., 2019). In addition, the mild conditions in the SFE process can alleviate the problems related to the thermal decomposition of components. SDE is the most used and classic method for extracting volatile components. Its principle is to distill the volatile components with steam, which is suitable for extracting volatile components that are stable in water and difficult to dissolve in water (Řebíčková et al., 2020). However, long-term and high-temperature extraction is easy to cause component hydrolysis and thermal degradation, and heat sensitive compounds are easy to be damaged (Pourmortazavi and Hajimirsadeghi, 2007). Therefore, from the point of view of the difference in principle between the two extraction processes, there may be two main reasons for the great difference in the AEO components extracted by the two methods. On the one hand, it is the influence of thermal stability. Steam distillation requires long-time azeotropy, which may lead to thermal decomposition of volatile components, resulting in low content of chromones in AEO samples (Yang, 2021). On the other hand, it is the influence of the difference in boiling points of components. The boiling points of 2- (2-phenylethyl) chromones are generally higher than those of sesquiterpenoids (Tian et al., 2019). Compared with SDE, SFE is easier to extract volatile components with high boiling points, which may be the reason that the content of chromones in AEO samples extracted by steam distillation is low, while that in AEO samples extracted by supercritical fluid is the opposite.

In addition, we found two peaks of plasticizer components in the chromatogram of S2 (Figure 2A). They were identified as diethyl phthalate (42.01%) and diethylhexyl phthalate (5.33%). 2-(2-phenylethyl) chromone derivatives and sesquiterpenes are the main characteristic components of AEOs, but the high content (about 5% and 42%, respectively) of the plasticizers should not exist. According to literatures (Wang et al., 2018), it is found that diethyl phthalate and diethylhexyl phthalate are not the proper components in AEOs. Phthalates is a kind of plasticizers often used in plastic products (Pecht et al., 2017; Luo et al., 2020). It is also common for illegal businesses to adulterate DEHP and other plasticizers into foods or essential oils to reduce the cost of products (Li and Chow, 2017). Studies (Mariana et al., 2016; Benjamin et al., 2017; Luo et al., 2020) have shown that excessive intake of these plasticizers will have adverse effects on human reproduction, development and cardiovascular system. Except for sample 2, AEO samples 3, 4, 5, 8, 14, 21, 22 and 23 also contain different contents of DEHP and diethyl phthalate. The samples with a plasticizer content of less than 1% may be due to be exposed to some plastic products during storage and packaging. According to literature (Pecht et al., 2017; Luo et al., 2020), the plasticizers may migrate from plastic containers to the loaded products as components in packaging materials. However, the plasticizer content in S2 accounts for about 47.34%, the most likely is the artificial addition of plasticizer for adulteration. Therefore, according to GC-MS analysis, it is considered that other AEO samples can be regarded as “authentic”, while S2 is adulterated. These information is very useful for evaluating the quality of AEOs and the practicality of Raman spectroscopy for distinguishing AEOs.

### 3.2 Raman spectrum analysis of AEOs

**FIGURE 4:**
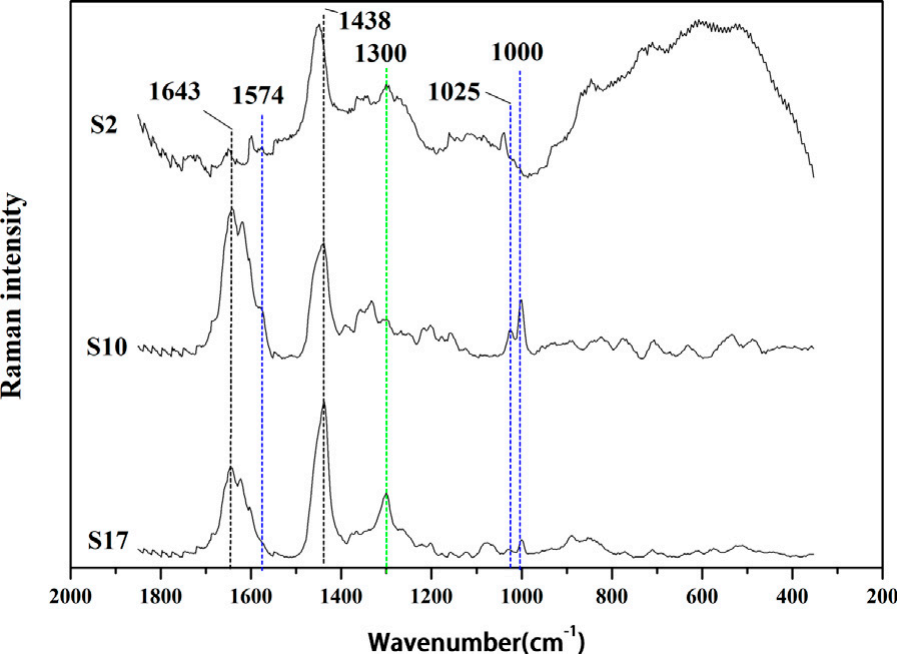
Raman spectrums of representative AEO samples: S2 (adulterated sample), S10 (extracted by SFE), S17 (extracted by SDE).

AEOs contain a wide variety of sesquiterpenoids and 2-(2-phenylethyl) chromones, and each component displays its own characteristic bands in its Raman spectrum, which can be assigned to contributions of the oil components to distinguish authentic AEOs from adulterated AEOs, and to classify different types of AEOs which may be related to their geographical origins or extraction methods. These “anomalies” can be observed by visual inspection of the spectrum and the application of multivariate analysis. By analyzing the Raman spectra of 30 AEOs, the Raman spectra of three samples (S2, S10 and S17) are shown as the representative Raman spectra of the samples in Figure 4. The Raman spectra of all 30 AEOs are shown in Supplementary Figures S1–S30 in Supplementary Material. Through Figure 4 and Supplementary Figures S1–S30, it can be found that the Raman spectra of 30 AEOs show some common bands, while different types of samples also show their own characteristic bands.

Since the Raman spectrum derives from the composition of the analyzed AEO samples, its important bands should be assigned in order to distinguish adulteration and category. All samples show similar peak intensities at the bands at 1,438 and 1,644 cm^−1^ although the sample two is weaker at these two bands. However, some significant differences among these Raman spectra were observed. For example, all the AEO samples extracted by SFE include characteristic Raman peaks at 1,574, 1,025 and 1,000 cm^−1^, while those extracted by SDE include a characteristic band at 1,300 cm^−1^. These unique Raman bands can be used to distinguish AEO samples made by different extraction methods. The common band at 1,644 cm^−1^ is attribute to C=C stretching mode (Schulz et al., 2002; Schulz and Baranska, 2007; Vargas Jentzsch et al., 2015; Vargas Jentzsch et al., 2018), and that at 1,438 cm^−1^ corrresponds to the CH_3_/CH_2_ bending modes (Vargas Jentzsch et al., 2015; Vargas Jentzsch et al., 2018; Cebi et al., 2021). The bands of 30 AEO samples at 1,644 and 1438cm^−1^ are some stronger and some weaker, which may be determined by the number of CH_3_ and CH_2_ groups and C=C bonds in the chemical components of different AEOs. And this is a reason that the two bands are not easy to be attributed to specific components, because most chemical components have such groups in their structures (Vargas Jentzsch et al., 2015). From the assignment of these two common bands, they may be contributed to the common components in authentic AEOs, such as agarospirol, benzylacetone, *etc.* The characteristic bands and assignment in the Raman spectra of the AEOs and standard reference materials are show in Table 3.

**TABLE 3:** The characteristic bands (cm^−1^) and assignment in the Raman spectra of the AEOs and their comparison with the Raman characteristics of standards and adulterants.

Wavenumbers of bands (cm-1)						
Sample17	Sample18	Sample2	2- (2-Phenylethyl) chromone	Diethyl phthalate	Diethylhexyl phthalate	Group and Vibrational Mode
1646 cm^−1^	1641 cm^−1^	—	1641 cm^−1^	—	—	v (C=C) stretching
—	1576 cm^−1^	—	1574 cm^−1^	1578 cm^−1^	1578 cm^−1^	carbon-carbon double bond ring quadrant stretching
1438 cm^−1^	1440 cm^−1^	—	—	—	—	δ(C-H) scissoring (Portarena et al., 2017; Portarena et al., 2019)
1300 cm^−1^	1300 cm^−1^ (weak)	1300cm^−1^ (weak)	—	—	—	δ(C-H) twisting (Portarena et al., 2017; Portarena et al., 2019)
—	1024 cm^−1^	—	1024 cm^−1^	—	—	C-C-O and C-O-C stretching mode (Lin-Vien et al., 1991; Schulz and Baranska, 2007)
—	1000 cm^−1^	—	1000 cm^−1^	—	—	Ring breathing (Si et al., 2019)

**FIGURE 5:**
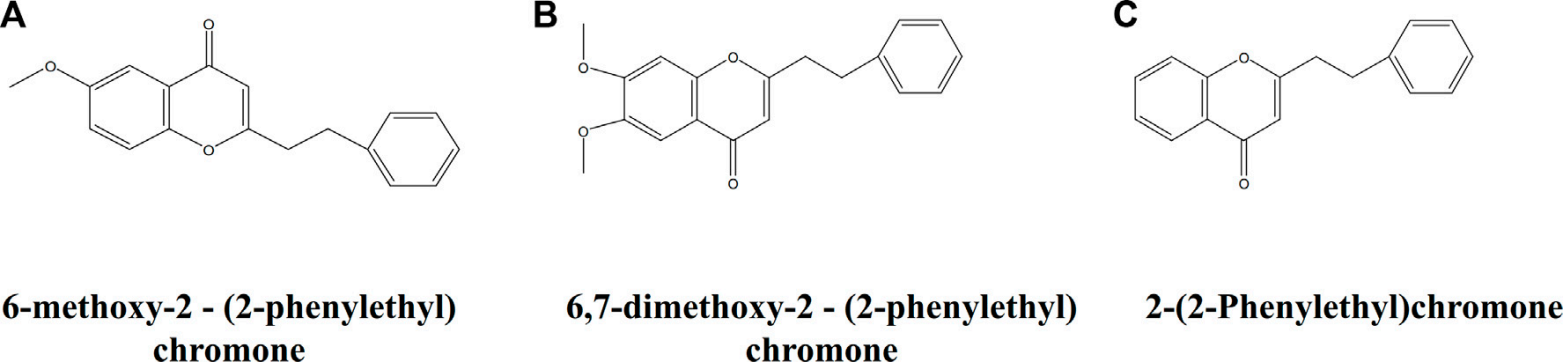
Molecular structure of three representative chromone derivatives. **(A)** 2-(2-Phenylethyl) chromone; **(B)** 6-methoxy-2 - (2-phenylethyl) chromone; **(C)** 6,7-dimethoxy-2 - (2-phenylethyl) chromone.

The AEOs obtained by SFE contain common characteristic bands at 1,574, 1025 and 1000 cm^−1^. The 1574 cm^−1^ band is assigned to the carbon-carbon double bond ring quadrant stretching mode of monosubstituted benzene (Júnior et al., 2016; Si et al., 2019), which is the characteristic group of 2- (2-phenylethyl) chromone derivatives (Figure 5), the iconic compounds in AEO. Therefore, this band is mainly attributed to chromone derivatives such as 2-(2-Phenylethyl) chromone, 6-methoxy-2 - (2-phenylethyl) chromone, 6,7-dimethoxy-2-(2-phenylethyl) chromone. The 1025 cm^−1^ band can be attributed to the out-of-phase C-C-O and C-O-C stretching mode (Lin-Vien et al., 1991; Schulz and Baranska, 2007; Chain et al., 2014), and the band of 1000 cm^−1^ is assigned to ring breathing vibration (Si et al., 2019). These two bands are considered as the most valuable Raman bands for identifying monosubstituted benzene. By analyzing the common features of the molecular structure formula of chromone derivatives in Figure 5, we can further confirm that these characteristic bands are contributed by chromone derivatives common to SFE samples. The AEO samples extracted by SDE contain a common strong band at 1300 cm^−1^, which may be attributed to C-H bending (twisting) (Júnior et al., 2016; Lee et al., 2018; Portarena et al., 2019) and mainly belongs to the structural characteristics of sesquiterpenes in AEOs.

**FIGURE 6:**
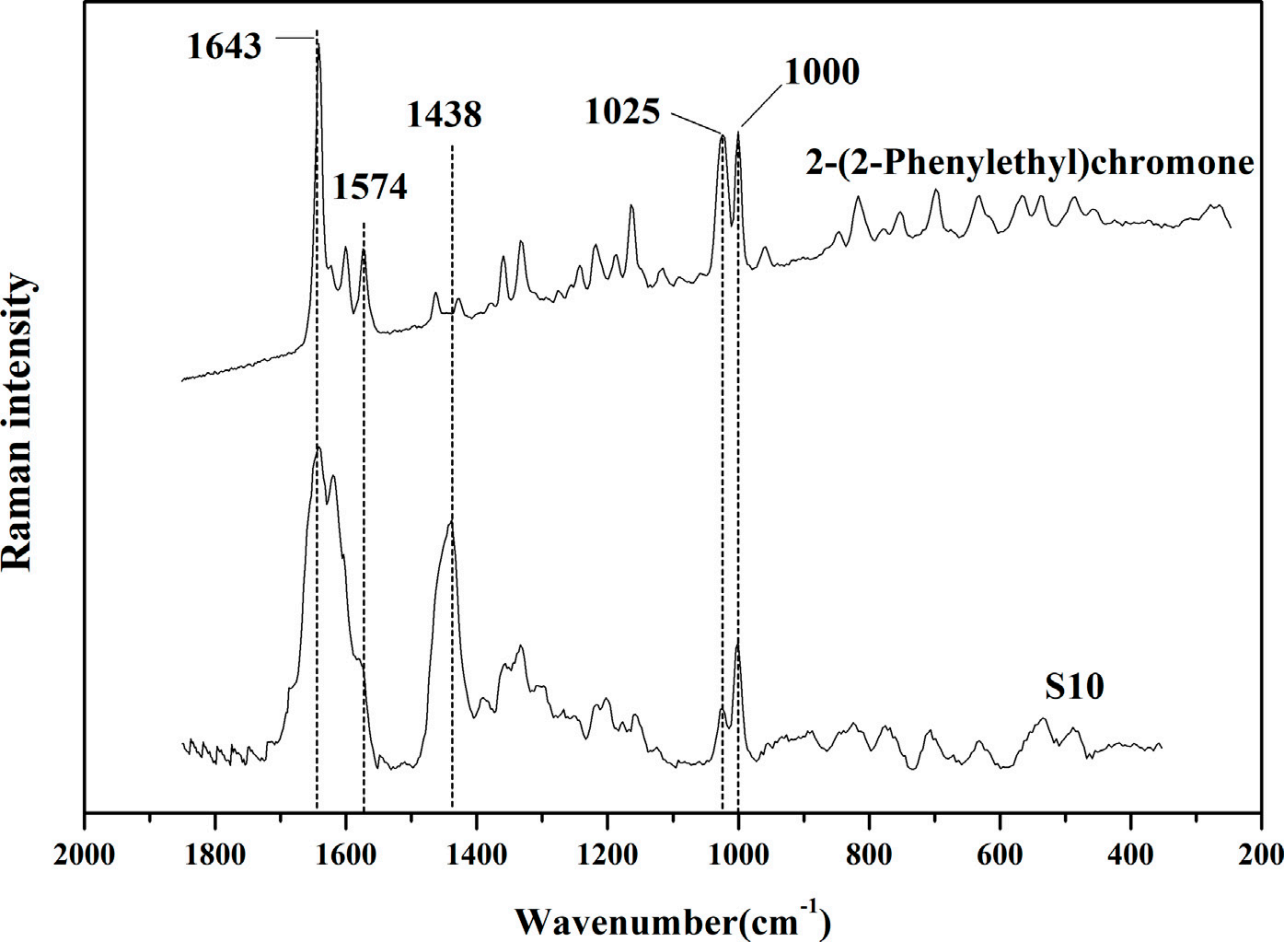
Comparison of Raman spectrums of S10 (representative sample of SFE) and 2-(2-Phenylethyl) chromone (standard reference material).

In order to further verify the correlation between the common Raman characteristic peaks of AEO samples extracted by SFE and their characteristic components, their common component 2- (2-phenylethyl) chromone was used as the reference material for Raman detection. It can be observed in Figure 6 that there are three characteristic bands with high intensity, 1574cm^−1^, 1025, 1000 cm^−1^, which are consistent with the characteristic bands in the 13 AEO samples extracted by SFE. Therefore, through the comparison of standard component, it can be almost confirmed that 1574, 1024c, 1000 cm^−1^ are the Raman characteristic bands of 2- (2-phenylethyl) chromone derivatives, which can be used for the identification of AEO samples extracted by SFE.

**FIGURE 7:**
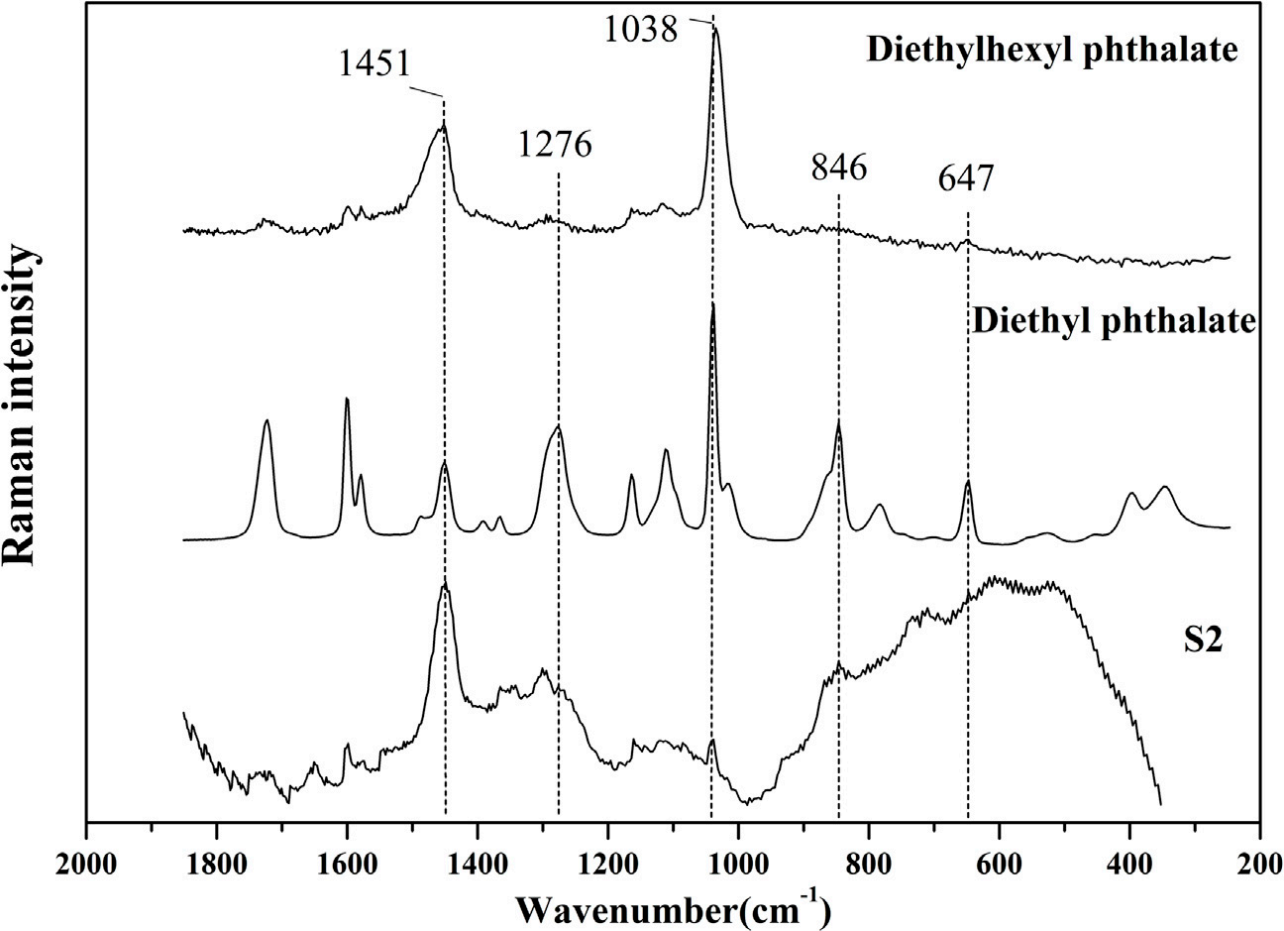
Comparison of Raman spectrums of S2 (adulterated sample) and diethyl phthalate, diethylhexyl phthalate (standard reference material of plasticizers).

It was found in the previous GC-MS analysis that S2 contained a high content of diethyl phthalate and diethylhexyl phthalate. It can be seen from Figure 7 that the main bands in the Raman spectrum of S2 include 1451, 1300,1276, 1038, 846 cm^−1^ and other bands. However, the characteristic bands of authentic AEOs such as 1,644 and 1574 cm^−1^ did not appear, so it is speculated that the above Raman bands in S2 are related to the adulterated components. Therefore, the reference materials of plasticizer components diethyl phthalate and diethylhexyl phthalate were used for Raman spectrum detection (Figure 7). The bands of 1,451, 1,276, 1,038 and 846 cm^−1^ in S2 also appeared in the standard of diethyl phthalate, and bands of 1,451 and 1,038 cm^−1^ appeared in the standard of diethylhexyl phthalate. These bands in the plasticizers are highly consistent with the Raman spectrum of S2, which further confirmed that Raman spectrum detection can characterize the adulteration of plasticizer in AEOs through rapid detection and characteristic spectrum, and the results are basically consistent with GC-MS analysis. The Raman spectra of the three reference materials in the spectral range of 1800-400 cm^−1^ are shown in Supplementary Figures S31–S33 in Supplementary Material.

### 3.3 Identification of classification and adulteration of AEOs by chemometrics

#### 3.3.1 Unsupervised pattern anlysis method of PCA

##### 3.3.1.1 Analysis of variance contribution rate and scores of AEO samples

**FIGURE 8:**
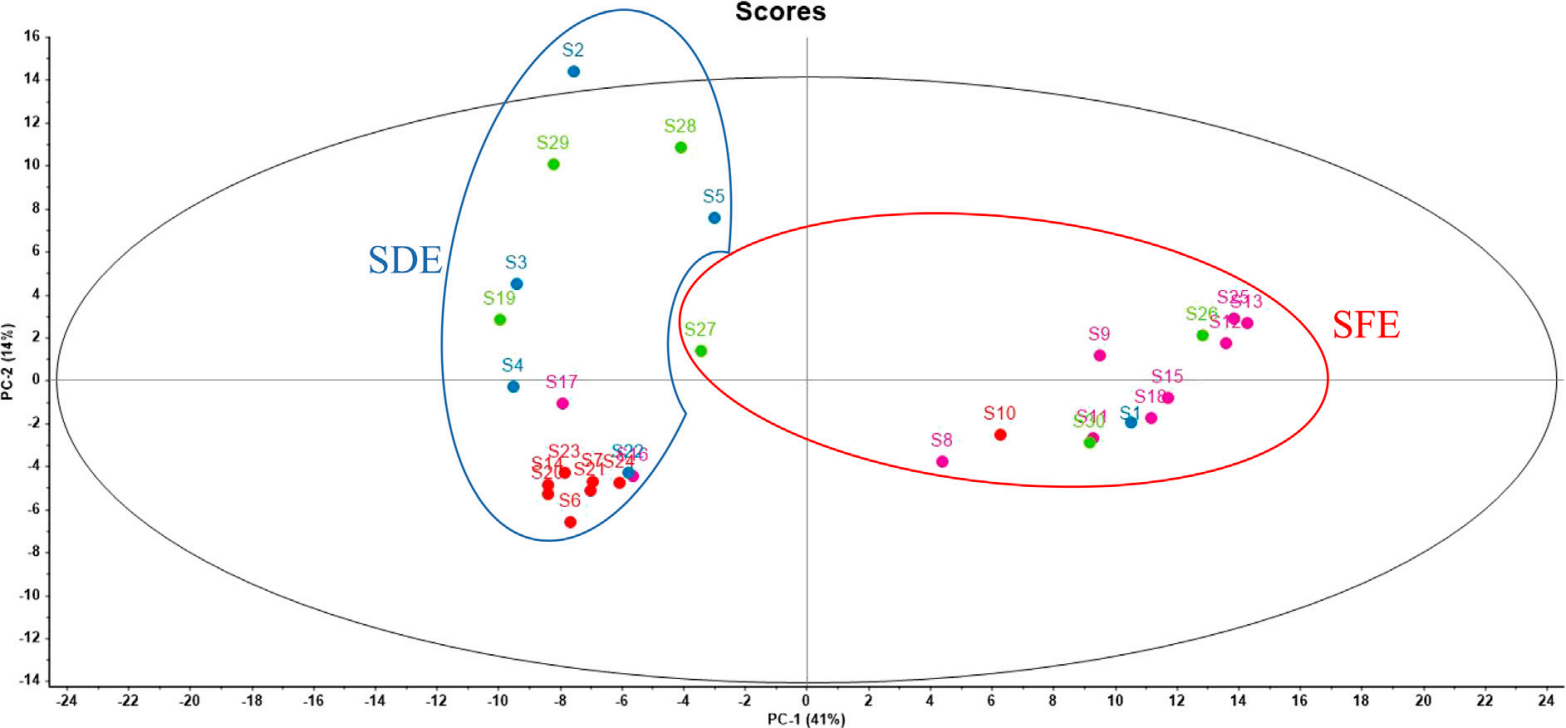
PCA scatter diagram of 30 AEOs. Each dot represents each sample (S1-S30). The origin is indicated by the color of the dots. Red dots: Hainan, China; Green dots: Guangxi, China; Rose red dots: Guangdong, China; Blue dots: Southeast Asian countries. The extraction method is represented by circle curve. Red circle: SFE samples; Blue circle: SDE samples.

PCA was performed on the Raman spectral data of 30 AEO samples, and then the obtained principal component scores were used to draw the scatter distribution diagram, so as to directly observe the distribution and aggregation of different data points in the two-dimensional diagram. The seven principal component scores with the highest values contributed 41.43%, 13.91%, 12.25%, 6.22%, 5.94%, 5.01%, 3.50% to the spectral information, respectively, and the cumulative contribution rate was 88.26%. A two-dimensional scatter plot was drawn with PC1 and PC2 as the *x* and *y* axes respectively, as shown in Figure 8. It is easier to classify the AEOs made by different processes by using PCA analysis, a typical chemometric method. The clustering formed by the samples can be observed by PCA analysis chart. The PCA scattering diagram of Raman spectrum data of all 30 AEOs is shown in Figure 8 with two groups clearly observed. Along the *X*-axis direction, on the scatter diagram of PC1, the AEOs extracted by SDE gather on the negative side, while the AEOs extracted by SFE gather on the positive side. It is worth noting that this clustering of Raman spectral data is based on differences in Raman spectral characteristics associated with the composition of biological components present in each AEO sample. The samples on the same side show that these samples have some common components, and some samples on the same side are far away from each other because there are some different components. It is also noted that among these samples, S27 is a SFE sample, but it is almost divided into the SDE group. We speculate that it may be the deviation of individual samples. In addition, since the origin and extraction method of the samples are provided by the supplier, it is also possible that the extraction method information of the sample is wrong, resulting in it not being correctly classified.

PCA results further proved the above differences in the Raman spectra of AEOs obtained from different geographical areas. In Figure 8, along the *X*-axis direction, on the scatter diagram of PC1, most of the AEOs from Guangdong gather on the positive side, while the AEOs from Hainan gather on the negative side. Along the *Y* axis, on the scatter plot of PC2, most of the AEOs from Hainan and Guangdong were concentrated in the central position or negative side, while the AEOs from southeast Asian countries such as Laos and Indonesia are scattered. There are differences in botanical origins between agarwood from China and Southeast Asian countries. Therefore, we speculate that this may be an important reason for the different in Raman spectral characteristics between these two types of AEOs. In addition, during the classification of geographical origins, we also found that one sample from Hainan (S10) and two samples from Guangdong (S16, S17) as outliers were not divided into the region of their geographical origin, but far away from other samples. We speculate that one of the reasons is that the number of tested samples is not rich enough, which may lead to individual errors. The second reason is that the same origin also contains a large area. For example, the underground of Guangdong also contains many refined small areas. The samples with large deviations in classification may be far away from other samples from the same origin in the refined area.

According to the analysis results, Raman spectroscopy can well distinguish the extraction methods of samples, and the main difference bands of the two extraction methods are obtained, and the analysis results are similar to those of GC-MS. However, in terms of geographical origin classification, due to the complex geographical areas and the samples obtained under two different extraction processes, the classification effect is relatively not good enough, and there are some deviations in individual samples. Among the 30 samples, Guangzhou, Hainan and Guangxi are the three largest geographical areas, and other origins mainly include Southeast Asian countries such as Malaysia and Laos. From the results of PCA analysis, most samples from Guangzhou, Hainan and Guangxi can basically be clearly divided from the position of the response surface, but several points are relatively abnormal data, which are obviously far away from other samples from the same origin. According to our analysis, there may be a variety of reasons leading to the deviation in the results of origin classification. The first reason is the number of samples. Although a total of 30 samples were selected for this test, the number of samples in each geographical area is relatively small, which may lead to individual deviations. The second reason is the classification deviation caused by different extraction methods, which may lead to some changes in the main components of essential oils due to their different extraction methods, and finally make the a SDE sample from Guangzhou more like the SFE sample in Hainan.

Among the 30 batches of collected AEO samples, it is identified by GC-MS detection that S2, S3, S4, S5, S8, S14, S21, S22 and S23 were added with different contents of plasticizer, namely “diethyl phthalate” and “diethylhexyl phthalate”. For those with a content of less than 1%, we speculate that some plastic products may be contacted during sample storage and processing, resulting in the residual plasticizer in the essential oil, but the plasticizer content in Sample two is as high as 5% and 42%, which is likely to be artificially added. PCA analysis of Raman data (Figure 8) shows that S2 is obviously separated from other samples and excluded from the region. In the original band of Raman spectrum (Supplementary Figures S1–S30), we can also clearly see that the Raman spectrum of S2 is significantly different from other samples, especially in the range of bands from 1000 to 400 cm^−1^. It shows that Raman spectroscopy can be used as a quality evaluation method for rapid identification of adulterated AEO samples.

##### 3.3.1.2 Loading diagram of principal components

**FIGURE 9:**
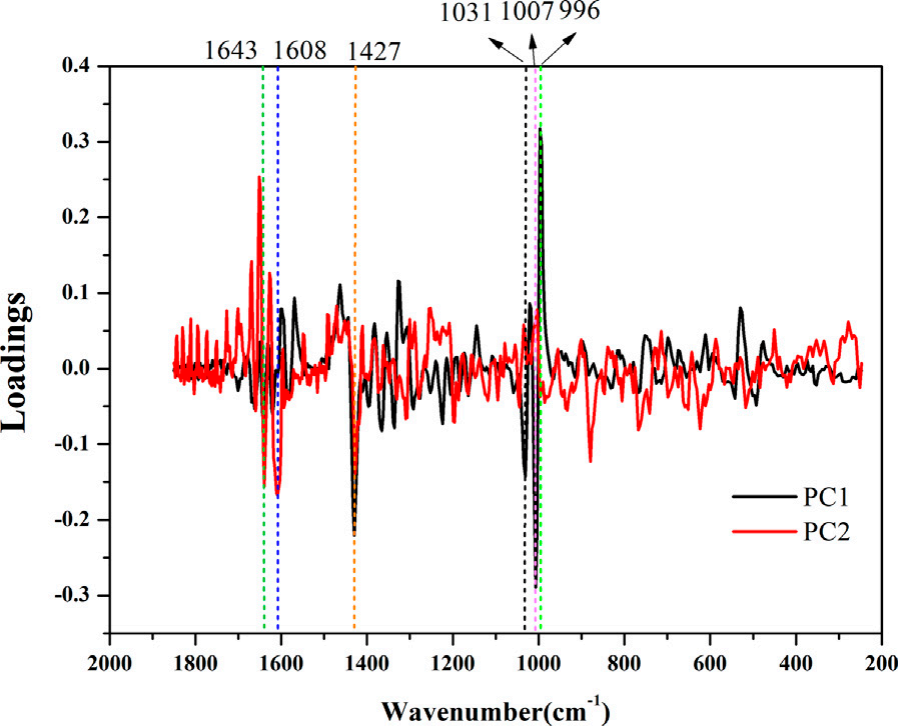
The loadings of the first two components for ordinary principal component analysis (PCA). The Raman bands at 1643^–1^, 1608^–1^, 1427^–1^, 1031^–1^, 1007^–1^, and 996 cm^−1^ showing large intragroup variations.

It can be inferred from the loading diagram (Figure 9) that it is not a single spectral band that affects the quality difference of AEOs, but the synergy of each characteristic spectral band in the Raman spectrum. In the loading diagram, the farther the spectral band is from the zero value of the ordinate, the greater the impact on the classification in the principal component analysis. It can be seen from the figure that the information of the first principal component is mainly from the characteristic spectral bands around 1007^−1^ and 1031^−1^, and the information of the second principal component is mainly from the characteristic spectral bands around 1643^−1^, 1608^−1^ and 1427^−1^. It shows that the classification and authentication of AEO samples by principal component analysis is mainly affected by these characteristic bands.

#### 3.3.2 Supervised pattern anlysis method of PLS-DA

##### 3.3.2.1 Establishment of classification and discrimination model

In order to identify AEO samples more accurately, PLS was used to establish the classification and discrimination model to distinguish the origins and extraction methods of AEO.

**FIGURE 10:**
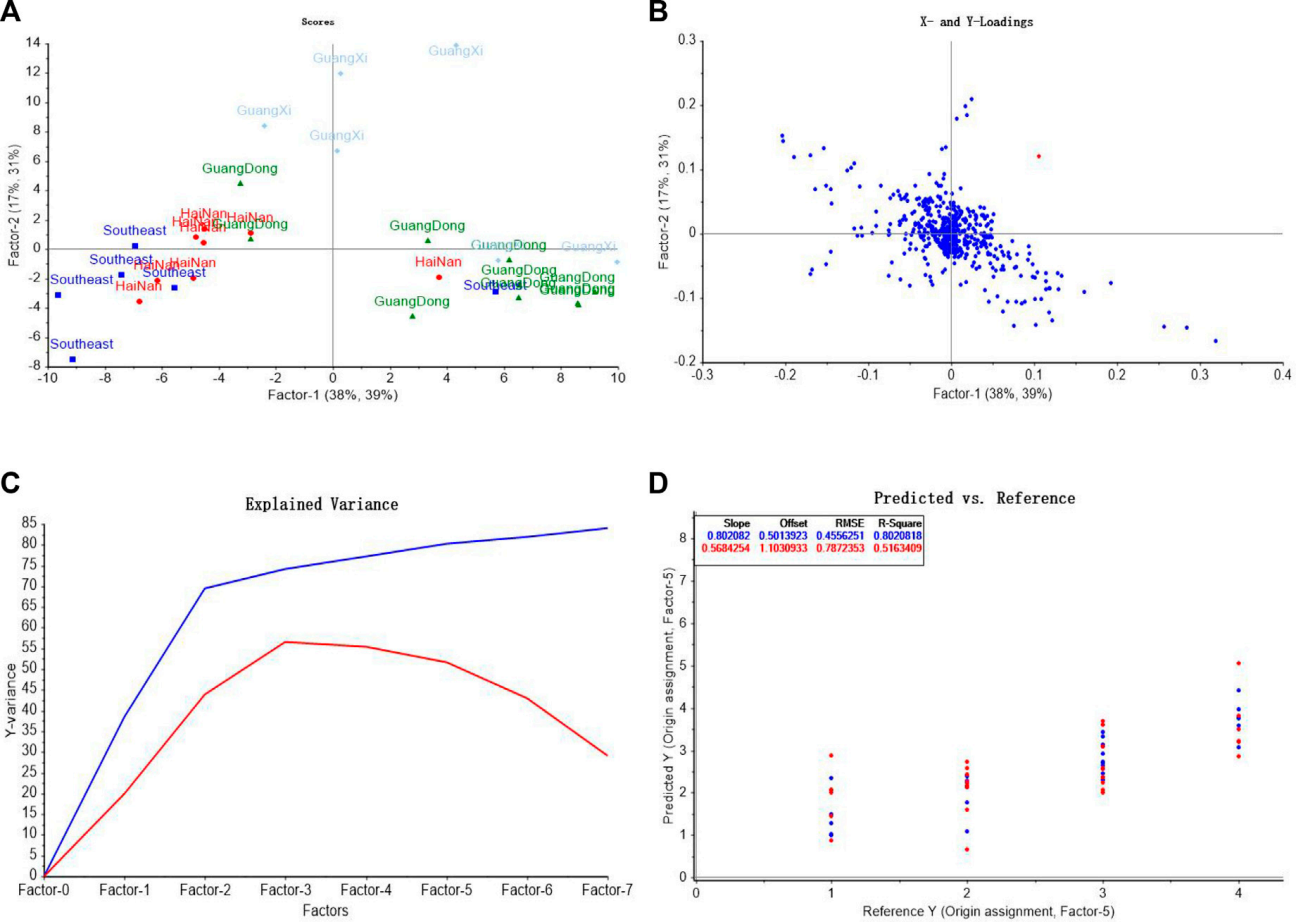
PLS-DA model analysis of different geographic origins of AEO samples. **(A)** PLS-DA score graph **(B)** The loadings of the first two components for PLS-DA; **(C)** Explained variance of factors in PLS-DA model **(D)** Comparison of predicted values and reference values of AEOs by PLS-DA.

In the discrimination model of origins, spectral data was taken as the input variable x, and the origin classification of Southeast Asia, Hainan, Guangdong and Guangxi was assigned as 1, 2, 3 and 4 respectively as the output variable y1, the score graph, loadings diagram, explained variance of factors, and comparison of predicted values and reference values were shown in Figures 10A–D. In order to select the appropriate quantity of principal components for modeling, the principal components were taken as the abscissa and the explained variance as the ordinate, as shown in Figure 10C. It can be seen from the figure that when the principal components reach 7, more than 80% of the variable data can be explained, so the seven principal components was selected for modeling. The origin of AEOs was predicted by PLS-DA, and the results are shown in Figure 10D. The predicted values of Southeast Asia samples were 0.75–2.17, Hainan samples were 1.68–2.60, Guangdong samples were 2.38–3.36, and Guangxi samples were 3.12–4.21, showing a relatively obvious clustering trend. Scatter diagram of predictive value and reference value of PLS-DA model is shown in Figure 10D. The correlation coefficient of the model is 0.802, indicating that the prediction results of the model can be used to distinguish the origins of AEOs. However, we also found that there were several samples that could not be accurately judged, indicating that the accuracy of the model still needs to be improved.

**FIGURE 11:**
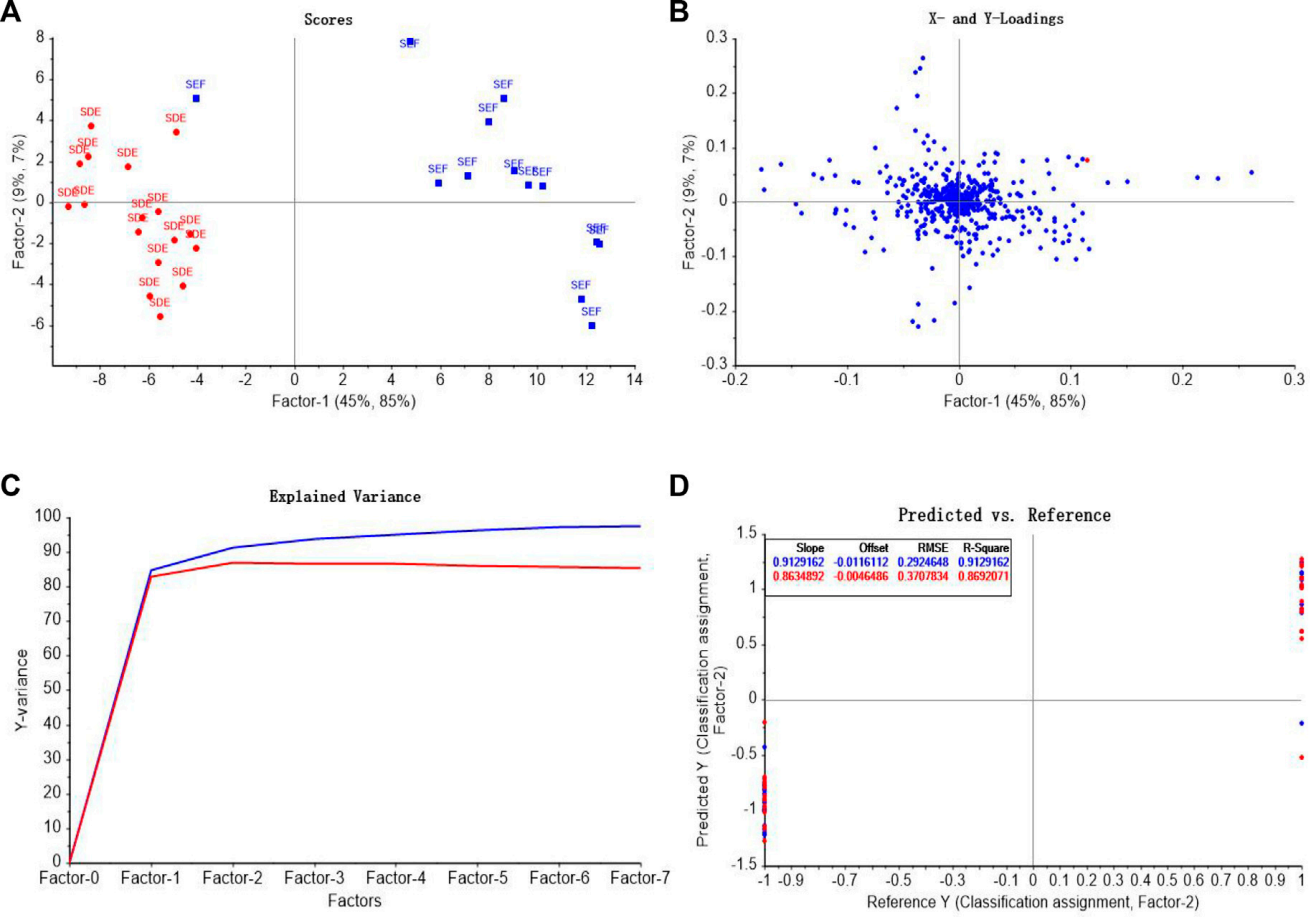
PLS-DA model analysis of different extraction methods of AEO samples. **(A)** PLS-DA score graph **(B)** The loadings of the first two components for PLS-DA; **(C)** Explained variance of factors in PLS-DA model **(D)** Comparison of predicted values and reference values of AEOs by PLS-DA.

In the discrimination model of different extraction methods, SFE and SDE were assigned as 1 and -1 as the output variable y2, respectively. Seven principal components were used for modeling, and the score graph, loadings diagram, explained variance of factors, and comparison of predicted values and reference values of AEOs were shown in Figures 11A–D. The predictive value of SFE samples was 0.61–1.19, and that of SDE samples was −1.24 to −0.57, showing a obvious clustering trend. Figure 11D is a scatter plot of the predictive value and reference value of the extraction method discrimination model. The correlation coefficient of the model is 0.913, which indicates that the model has high accuracy and can make good judgments on samples with different extraction methods.

##### 3.3.2.2 Model validation

The applicability of the model for origin discrimination was verified through unknown samples in the validation data subset (2 samples for each origin, eight samples in total). The comparison between the predicted values and the assigned values was shown in Table 4. Among them, the actual origin of S3 and S5 is Southeast Asia, and the predicted value range of the model is 0.81–1.10, which conforms to the assigned value range of 1 ± 0.5. The origin of S20 and S21 samples is Hainan, and the predicted value range of the model is 2.17–2.18, which is within the assigned value range of 2 ± 0.5. The origin of S9 and S25 is Guangdong, and the predicted value ranges from 2.88 to 3.09, which conforms to the assigned value of 3 ± 0.5. The origin of S26 and S27 is Guangxi, and the predicted value range is 3.87–4.12, which is within the assigned value range of 4 ± 0.5. From the prediction results, it can be seen that the origin of the eight samples from validation set can be correctly identified, which proves that Raman spectroscopy combined with PLS-DA method can be used to identify AEOs from fdifferent origins.

**TABLE 4:** Predicted results of the PLS discriminant model in validation samples.

No.	Classification information	Sample information	Predicted values	Assigned values
1	Origin classification	S3 (Southeast Asia)	1.102,308	1
2		S5 (Southeast Asia)	0.810,964	1
3		S20 (Hainan, China)	2.173,878	2
4		S21 (Hainan, China)	2.182,529	2
5		S9 (Guangdong, China)	3.091751	3
6		S25 (Guangdong, China)	2.882,586	3
7		S26 (Guangxi, China)	4.116,474	4
8		S27 (Guangxi, China)	3.875,032	4
9	Extraction method classification	S14 (SFE)	−1.188,087	-1
10		S20 (SFE)	−0.9986625	-1
11		S13 (SDE)	0.956,342	1
12		S15 (SDE)	0.9255514	1

To verify the applicability of the model for the identification of extraction methods, the unknown samples in the validation data subset (2 samples for each extraction method, four samples in total) were verified. The comparison between the predicted values and the assigned values is shown in Table 4. The extraction method of S14 and S20 is SFE, and the predictive value range is −1.18 to −0.99, which conforms to the assignment interval of - 1 ± 0.5. S13 and S15 is extracted by SDE, and the predictive value range is 0.92–0.96, which is within the assigned value of 1 ± 0.5. The extraction methods of four unknown AEO samples can be correctly identified, which proves that Raman spectroscopy combined with PLS-DA can identify the extraction methods of AEOs.

## 4 Discussion

As a valuable aromatic traditional Chinese medicine, AEO has many geographical origins, various extraction methods and serious adulteration in the market. Its quality evaluation and authenticity identification have been widely concerned. Therefore, Raman spectroscopy combined with GC-MS and PCA were used to evaluate the quality of AEOs. The aim of this study is to provide a novel and convenient method for the rapid analysis and discrimination of AEOs. The results of GC-MS analysis showed that the content and composition of AEOs were greatly affected by the extraction method, and the results of Raman spectroscopy combined with PCA analysis were consistent with those of GC-MS. Raman characteristic spectrum can completely distinguish the extraction methods of 30 AEO samples, but in terms of origin classification, due to the complexity of geographical areas and the two different extraction processes, the classification effect is relatively general, and individual samples have deviation.

In terms of extraction methods, the 30 samples are mainly divided into SDE and SFE. Those AEO samples using SFE include S1, S8-S13, S15, S18, S25, S26, S27 and S30. According to the PCA classification results, the Raman spectra of the studied 30 AEOs can be divided into two groups according to their differences, and the two parts are clearly separated. However, among the 13 SFE samples, S27 is an exception. Although it is a sample extracted by SFE, it is similar to the sample extracted by SDE. We further analyzed the original spectrum of S27 (Supplementary Material, Figure S27) and found that the main bands with high intensity were at 1653, 1622 1438 1300 and 1000 cm^−1^. As we analyzed in the part of characteristic band assignment, the main characteristic bands of AEOs extracted by SFE are located at 1574, 1025 and 1000 cm^−1^, while the SDE samples generally have high intensity peaks at 1300 cm^−1^. S27 has a high intensity peak at 1300 cm^−1^, which is the biggest difference between it and other SFE samples. This may be the main reason for its deviation in PCA classification and being mixed in SDE samples.

In terms of geographical origin, 30 samples were divided into four main geographical areas including Guangdong (China), Hainan (China), Guangxi (China) and Southeast Asian countries (Cambodia, Indonesia, Laos, Thailand). In the scatter chart composed of principal component scores PC1 and PC2, the scatter points representing Hainan and Guangdong were gathered together, closely distributed and occupying a relatively small space, which reflects the high similarity of components between different batches of AEOs from the same origin in the above two types. However, the data points representing Guangxi and Southeast Asia were scattered in a large range, indicating that the composition of different batches of AEOs from these two types of origins is quite different. The samples from Guangdong include S8, S9, S11–13, S15-S18 and S25. These samples are roughly divided into two regions, most of which are concentrated on the right side of the *X*-axis of PCA analysis, but S16 and S17 are significantly different from other samples and are divided on the left side of the *X*-axis in PCA analysis. In order to clarify the reasons for this difference, we analyzed the detailed information of these two samples, and it is found that they were extracted by SDE, while other AEO samples from Guangdong were extracted by SFE. This indicates that different extraction methods lead to obvious differences between these two samples and other samples from the same origin. Further analysis of the original spectra of S16 and S17 shows that the main bands with high intensity located at 1300cm^−1^, which is the characteristic bands of SDE samples, while the high intensity bands of other Guangdong AEO samples mainly include SFE characteristic bands of 1574 m^−1^ and 1000 cm^−1^. The samples from Hainan include S6-S7, S10, S14, S20-S21 and S23-S24. Most of these samples are concentrated on the left side of the *X*-axis of PCA analysis, but S10 is obviously different from other samples and is divided on the right side. This may be because it is a sample extracted by SFE. The samples from Guangxi include S19 and S26-S30. These samples are roughly divided into two regions, most of which are concentrated on the left side of the *X*-axis of PCA analysis, but S26 and S30 are significantly different from other samples and are divided on the right side. Through the analysis of the classification results of Guangxi samples, it is found that the AEO samples in Guangxi have a large degree of deviation from each other, unlike Hainan samples, which have a high degree of aggregation in classification. This indicates that the AEOs from Guangxi have relatively large differences in composition. Other samples (S1-S5 and S22) are mainly come from Southeast Asian countries. We found that the samples from such countries are also far apart from each other, indicating that there are large differences in their compositions. To a certain degree, this result reflects that Guangdong and Hainan, as the main production areas of AEOs, have more systematic and standardized cultivation, production and processing technologies for this product, which leads to the high similarity of chemical components contained in different batches of samples. On the contrary, the quality uniformity of AEOs from Southeast Asia and Guangxi is slightly inferior.

From the perspective of authenticity identification, the Raman spectrum of adulterated sample two is obviously different from other samples and is separated on the PCA analysis diagram. By analyzing the original spectrum of S2, it is found that the main bands with high intensity were at 1449 cm^−1^. Through the analysis of adulterated samples, it can be confirmed that Raman spectrum has a good identification degree for adulterated samples, which indicates that this method can have a broad application prospect in the identification of the authenticity of AEOs. In addition, one of the most important highlights is that we have confirmed the characteristic bands of the plasticizer diethyl phthalate and diethylhexyl phthalate, including 1451, 1276, 1038, 846 and 647 cm^−1^, which are not found in genuine AEOs. Therefore, if these characteristic bands are found in the commercial AEOs at the same time and have high intensity, we have reason to quickly judge that they may be adulterated by adding plasticizers.

In general, Raman spectroscopy can be used as an effective method for the quality evaluation of AEOs. It will also provide a reference for developing fast, economical and effective method of Raman spectroscopy for qualitative and quantitative monitoring of other essential oils.

## 5 Conclusion

The application of Raman spectroscopy to the determination of chemical constituents of AEOs is helpful for the qualitative monitoring of different extraction methods, different geographical origins, adulterated samples and their chemical composition, so as to lay a foundation for developing a fast, economical and effective method for analyzing AEOs. Raman spectroscopy and PCA were used to evaluate and characterize the 30 batches of AEOs. The characteristic Raman spectra corresponding to each AEO sample were obtained, and the characteristic component of AEOs, chromone derivatives, and two commonly used adulterants were also detected by Raman spectroscopy. This not only provides a basis and effective method for the quality evaluation and authenticity identification of AEOs in the market, but also provides a reference for developing novel method for qualitative and quantitative monitoring of other essential oils.

## Data Availability

The original contributions presented in the study are included in the article/Supplementary Material, further inquiries can be directed to the corresponding authors.

## References

[B1] Abu-Darwish D. Shibli R. Al-Abdallat A. M. (2022). *In vitro* cultures and volatile organic compound production in chiliadenus montanus (vhal.) brullo. Plants (Basel). 11 (10), 1326. 10.3390/plants11101326 35631753 PMC9148159

[B2] Ben Hassine D. Kammoun El Euch S. Rahmani R. Ghazouani N. Kane R. Abderrabba M. (2021). Clove buds essential oil: The impact of grinding on the chemical composition and its biological activities involved in consumer’s health security. BioMed Res. Int. 2021, 9940591. 10.1155/2021/9940591 34381841 PMC8352679

[B3] Benjamin S. Masai E. Kamimura N. Takahashi K. Anderson R. C. Faisal P. A. (2017). Phthalates impact human health: Epidemiological evidences and plausible mechanism of action. J. Hazard. Mater. 340, 360–383. 10.1016/j.jhazmat.2017.06.036 28800814

[B4] Camele I. Gruľová D. Elshafie H. S. (2021). Chemical composition and antimicrobial properties of Mentha× piperita cv.‘Kristinka’essential oil. Plants 10 (8), 1567. 10.3390/plants10081567 34451612 PMC8399209

[B5] Cebi N. Arici M. Sagdic O. (2021). The famous Turkish rose essential oil: Characterization and authenticity monitoring by FTIR, Raman and GC–MS techniques combined with chemometrics. Food Chem. 354, 129495. 10.1016/j.foodchem.2021.129495 33743448

[B6] Chain F. Romano E. Leyton P. Paipa C. Catalán C. A. N. Fortuna M. A. (2014). An experimental study of the structural and vibrational properties of sesquiterpene lactone cnicin using FT-IR, FT-Raman, UV–visible and NMR spectroscopies. J. Mol. Struct. 1065, 160–169. 10.1016/j.molstruc.2014.02.057

[B7] Chen S. Rui R. Wang S. He X. (2022). Comparative analysis of the floral fragrance compounds of panax notoginseng flowers under the panax notoginseng-pinus agroforestry system using SPME-GC-MS. Molecules 27 (11), 3565. 10.3390/molecules27113565 35684502 PMC9182305

[B8] Chen X. Zhu X. Feng M. Zhong Z. Zhou X. Chen X. (2017). Relationship between expression of chalcone synthase genes and chromones in artificial agarwood induced by formic acid stimulation combined with Fusarium sp. A2 inoculation. Molecules 22 (5), 686. 10.3390/molecules22050686 28441359 PMC6154532

[B9] Costa E. V. de Souza C. A. Galvão A. F. Silva V. R. Santos L. D. S. Dias R. B. (2022). Duguetia pycnastera sandwith (annonaceae) leaf essential oil inhibits HepG2 cell growth *in vitro* and *in Vivo* . Molecules 27 (17), 5664. 10.3390/molecules27175664 36080430 PMC9458038

[B10] Geng T. (2021). Study on extraction and biological activity of agarwood essential oil. Chongqing, China: Southwest University. 10.27684/d.cnki.gxndx.2020.003459

[B11] Han Q. Yang S. Chen X. Zhong Z. Zhou X. Zhang W. (2019). Analysis and comparison of chemical composition of agarwood volatile oil.Journal of Chinese Medicinal Materials. J. Chin. Med. Mater. 42 (07), 1566–1571. 10.13863/j.issn1001-4454.2019.07.022

[B12] Hashim Y. Z. H. Y. Kerr P. G. Abbas P. Salleh H. M. (2016). Aquilaria spp.(agarwood) as source of health beneficial compounds: A review of traditional use, phytochemistry and pharmacology. J. Ethnopharmacol. 189, 331–360. 10.1016/j.jep.2016.06.055 27343768

[B13] Heidary Jamebozorgi F. Yousefzadi M. Firuzi O. Nazemi M. Jassbi A. R. (2019). *In vitro* anti-proliferative activities of the sterols and fatty acids isolated from the Persian Gulf sponge; Axinella sinoxea. DARU J. Pharm. Sci. 27 (1), 121–135. 10.1007/s40199-019-00253-8 PMC6593024 30887402

[B14] Hidayat W. Shakaff A. Y. M. Ahmad M. N. Adom A. H. (2010). Classification of agarwood oil using an electronic nose. Sensors 10 (5), 4675–4685. 10.3390/s100504675 22399899 PMC3292139

[B15] Hu R. He T. Zhang Z. Yang Y. Liu M. (2019). Safety analysis of edible oil products via Raman spectroscopy. Talanta 191, 324–332. 10.1016/j.talanta.2018.08.074 30262067

[B16] Jaradat N. Hawash M. Qadi M. Abualhasan M. Odetallah A. Qasim G. (2022). Chemical markers and pharmacological characters of Pelargonium graveolens essential oil from Palestine. Molecules 27 (17), 5721. 10.3390/molecules27175721 36080486 PMC9457828

[B17] Jerković I. Marijanović Z. Gugić M. Roje M. (2011). Chemical profile of the organic residue from ancient amphora found in the Adriatic Sea determined by direct GC and GC-MS analysis. Molecules 16 (9), 7936–7948. 10.3390/molecules16097936 22143551 PMC6264281

[B18] Júnior P. H. R. de Sá Oliveira K. de Almeida C. E. R. De Oliveira L. F. C. Stephani R. da Silva Pinto M. (2016). FT-Raman and chemometric tools for rapid determination of quality parameters in milk powder: Classification of samples for the presence of lactose and fraud detection by addition of maltodextrin. Food Chem. 196, 584–588. 10.1016/j.foodchem.2015.09.055 26593531

[B19] Lee J. Y. Park J. H. Mun H. Shim W. B. Lim S. H. Kim M. G. (2018). Quantitative analysis of lard in animal fat mixture using visible Raman spectroscopy. Food Chem. 254, 109–114. 10.1016/j.foodchem.2018.01.185 29548429

[B20] Li H. Li Y. Zhang X. Ren G. Wang L. Li J. (2021). The combination of Aquilaria sinensis (Lour.) Gilg and Aucklandia costus Falc. volatile oils exerts antidepressant effects in a CUMS-induced rat model by regulating the HPA Axis and levels of neurotransmitters. Front. Pharmacol. 11, 614413. 10.3389/fphar.2020.614413 33716727 PMC7943885

[B21] Li W. C. Chow C. F. (2017). Adverse child health impacts resulting from food adulterations in the Greater China Region. J. Sci. Food Agric. 97 (12), 3897–3916. 10.1002/jsfa.8405 28466508

[B22] Lin-Vien D. Colthup N. B. Fateley W. G. Grasselli J. G. (1991). The handbook of infrared and Raman characteristic frequencies of organic molecules. Elsevier.

[B23] Luo Q. Liu Z. H. Yin H. Dang Z. Wu P. X. Zhu N. W. (2020). Global review of phthalates in edible oil: An emerging and nonnegligible exposure source to human. Sci. Total Environ. 704, 135369. 10.1016/j.scitotenv.2019.135369 31812395

[B24] Mariana M. Feiteiro J. Verde I. Cairrao E. (2016). The effects of phthalates in the cardiovascular and reproductive systems: A review. Environ. Int. 94, 758–776. 10.1016/j.envint.2016.07.004 27424259

[B25] Omar J. Sarmiento A. Olivares M. Alonso I. Etxebarria N. (2012). Quantitative analysis of essential oils from rosemary in virgin olive oil using Raman spectroscopy and chemometrics. J. Raman Spectrosc. 43 (8), 1151–1156. 10.1002/jrs.3152

[B26] Pecht M. G. Ali I. Carlson A. (2017). Phthalates in electronics: The risks and the alternatives. Ieee Access 6, 6232–6242. 10.1109/access.2017.2778950

[B27] Persoon G. A. Beek H. (2008). “Growing ‘the wood of the gods’: Agarwood production in Southeast Asia,” in Smallholder tree growing for rural development and environmental services (Dordrecht: Springer), 245–262.

[B28] Portarena S. Anselmi C. Zadra C. Farinelli D. Famiani F. Baldacchini C. (2019). Cultivar discrimination, fatty acid profile and carotenoid characterization of monovarietal olive oils by Raman spectroscopy at a single glance. Food control. 96, 137–145. 10.1016/j.foodcont.2018.09.011

[B29] Portarena S. Baldacchini C. Brugnoli E. (2017). Geographical discrimination of extra-virgin olive oils from the Italian coasts by combining stable isotope data and carotenoid content within a multivariate analysis. Food Chem. 215, 1–6. 10.1016/j.foodchem.2016.07.135 27542443

[B30] Pourmortazavi S. M. Hajimirsadeghi S. S. (2007). Supercritical fluid extraction in plant essential and volatile oil analysis. J. Chromatogr. A 1163 (1-2), 2–24. 10.1016/j.chroma.2007.06.021 17624357

[B31] Řebíčková K. Bajer T. Šilha D. Ventura K. Bajerová P. (2020). Comparison of chemical composition and biological properties of essential oils obtained by hydrodistillation and steam distillation of Laurus nobilis L. Plant Foods Hum. Nutr. 75 (4), 495–504. 10.1007/s11130-020-00834-y 32710382

[B32] Rodríguez-Solana R. Daferera D. J. Mitsi C. Trigas P. Polissiou M. Tarantilis P. A. (2014). Comparative chemotype determination of Lamiaceae plants by means of GC–MS, FT-IR, and dispersive-Raman spectroscopic techniques and GC-FID quantification. Industrial Crops Prod. 62, 22–33. 10.1016/j.indcrop.2014.08.003

[B33] Schulz H. Baranska M. (2007). Identification and quantification of valuable plant substances by IR and Raman spectroscopy. Vib. Spectrosc. 43 (1), 13–25. 10.1016/j.vibspec.2006.06.001

[B34] Schulz H. Schrader B. Quilitzsch R. Steuer B. (2002). Quantitative analysis of various citrus oils by ATR/FT-IR and NIR-FT Raman spectroscopy. Appl. Spectrosc. 56 (1), 117–124. 10.1366/0003702021954296

[B35] Si M. Z. Li L. Zhang C. Y. Zhang D. Q. Li J. W. Yang Y. G. (2019). Situ research on fresh kaempferia galanga L. And kaempferia rotunda L. Oil cells with Raman spectroscopy. Chin. J. Trop. Crops 40 (9), 1817–1822. 10.3969/j.issn.1000-2561.2019.09.021

[B36] Su Y. Mao D. S. Qu R. F. Li Z. Y. Mao D. B. (2012). Analysis of volatile components in fenugreek extract (Trigonella foenum-graecum L) by stir bar sorptive extraction-thermal desorption and gas chromatography-mass spectrometry. Sci. Technol. Food Industry 33 (13), 78–80+83. 10.13386/j.issn1002-0306.2012.13.017

[B37] Tian C. P. Song Y. L. Xu, H. T. Niu S. Q. Wu Z. H. Shen L. Q. (2019). Composition analysis, antioxidative and antibacterial activities comparison of agarwood oils extracted by supercritical and stem distillation. China J. Chin. Materia Medica 44 (18), 4000–4008. 10.19540/j.cnki.cjcmm.20190629.302 31872737

[B38] Vargas Jentzsch P. Gualpa F. Ramos L. A. Ciobotă V. (2018). Adulteration of clove essential oil: Detection using a handheld Raman spectrometer. Flavour Fragr. J. 33 (2), 184–190. 10.1002/ffj.3438

[B39] Vargas Jentzsch P. Ramos L. A. Ciobotă V. (2015). Handheld Raman spectroscopy for the distinction of essential oils used in the cosmetics industry. Cosmetics 2 (2), 162–176. 10.3390/cosmetics2020162

[B40] Velioglu S. D. Temiz H. T. Ercioglu E. Velioglu H. M. Topcu A. Boyaci I. H. (2017). Use of Raman spectroscopy for determining erucic acid content in canola oil. Food Chem. 221, 87–90. 10.1016/j.foodchem.2016.10.044 27979286

[B41] Wang M. R. Li W. Luo S. Zhao X. Ma C. H. Liu S. X. (2018). GC-MS study of the chemical components of different Aquilaria sinensis (lour.) gilgorgans and agarwood from different asian countries. Molecules 23 (9), 2168. undefined. 10.3390/molecules23092168 30154355 PMC6225301

[B42] Yang J. I. Mei W. I. Dong W. H. Dai H. F. (2016). Identification of two batches of fake agarwoods. Asia-Pacific Tradit. Med. 23, 35–39. 10.11954/ytctyy.201623014

[B43] Yang Y. S. (2021). Major extraction techniques, applications and research progress of plant essential oils. Mod. Chem. Res. 04, 153–154.

[B44] Yao C. Qi L. Zhong F. Li N. Ma Y. (2022). An integrated chemical characterization based on FT-NIR, GC–MS and LC-MS for the comparative metabolite profiling of wild and cultivated agarwood. J. Chromatogr. B 1188, 123056. 10.1016/j.jchromb.2021.123056 34871920

[B45] Yousefi M. Rahimi-Nasrabadi M. Pourmortazavi S. M. Wysokowski M. Jesionowski T. Ehrlich H. (2019). Supercritical fluid extraction of essential oils. TrAC Trends Anal. Chem. 118, 182–193. 10.1016/j.trac.2019.05.038

