# Supplementary material for: An integrated approach utilizing raman spectroscopy and chemometrics for authentication and detection of adulteration of agarwood essential oils
Source: Front Chem. 2022 Dec 21;10:1036082. doi: 10.3389/fchem.2022.1036082 (PMC9810987; doi:10.3389/fchem.2022.1036082)
Supplement: Supplementary file 1 [file DataSheet1.PDF]

Supplementary Material

1.1 Supplementary Figures

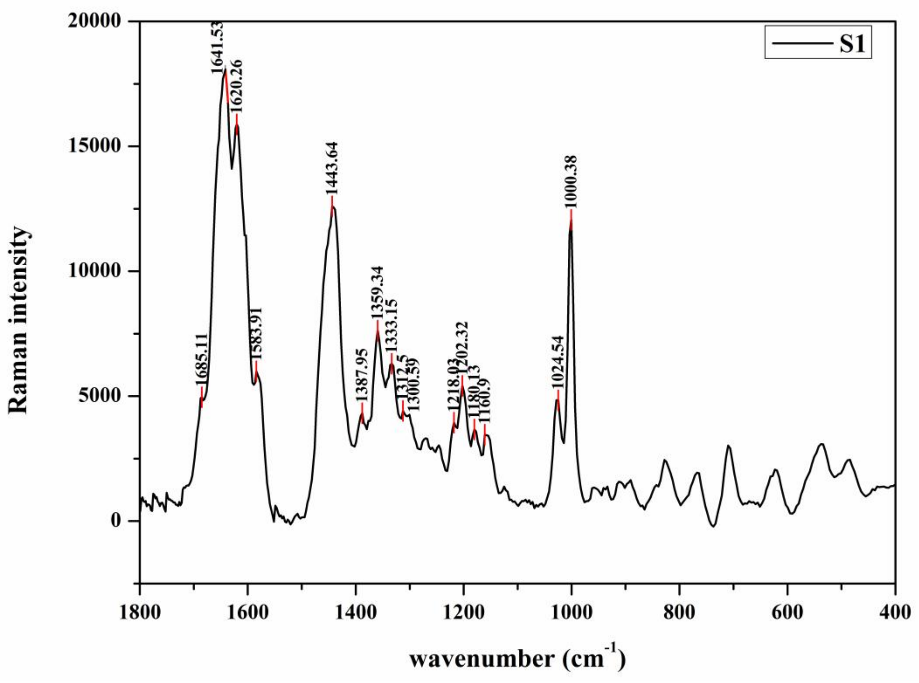

Figure S1. Raman spectrum of S1.

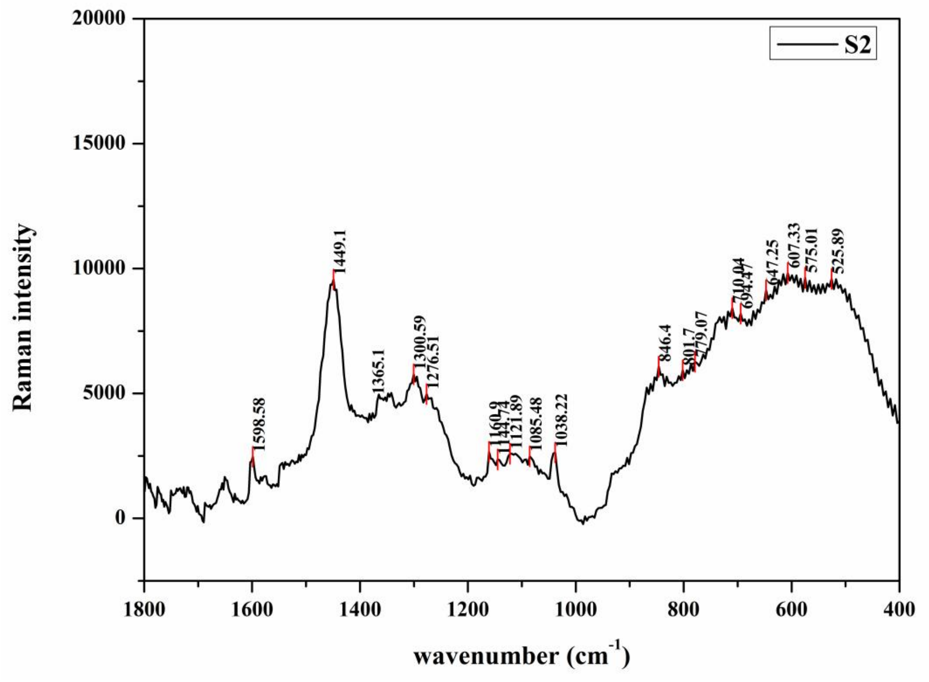

Figure S2. Raman spectrum of S2.

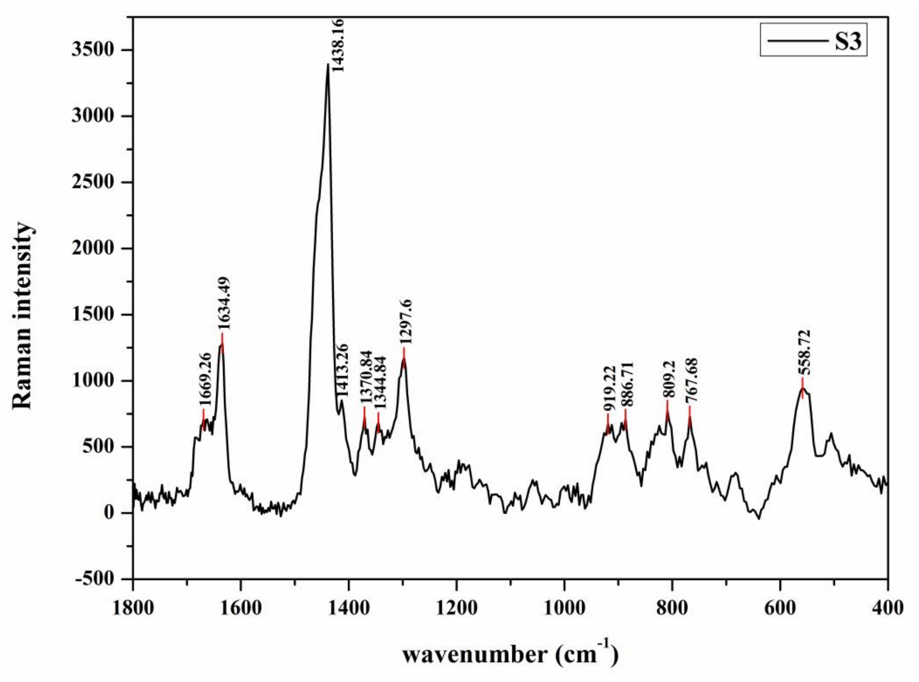

Figure S3. Raman spectrum of S3.

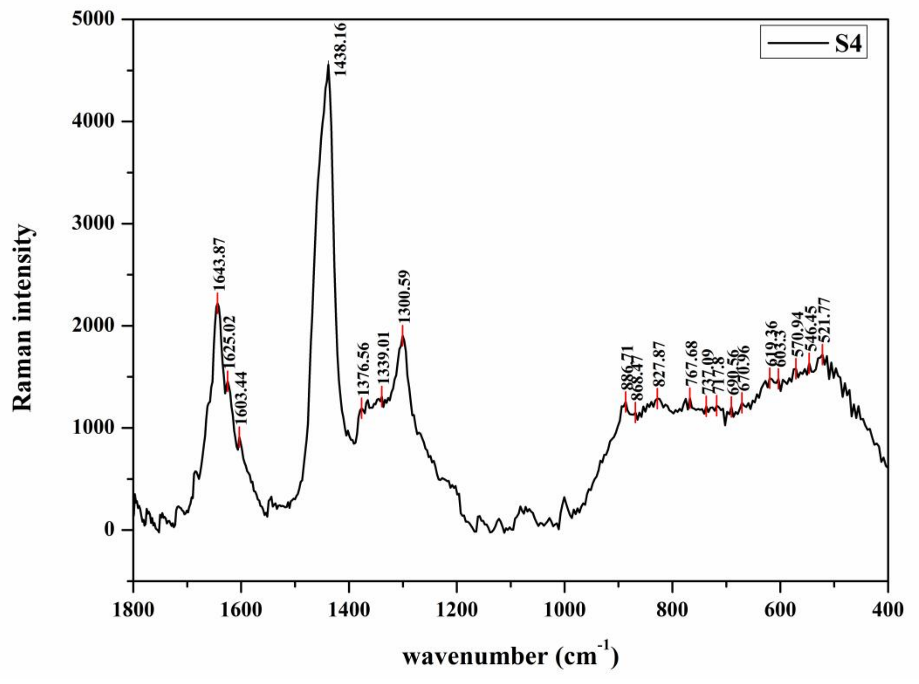

Figure S4. Raman spectrum of S4.

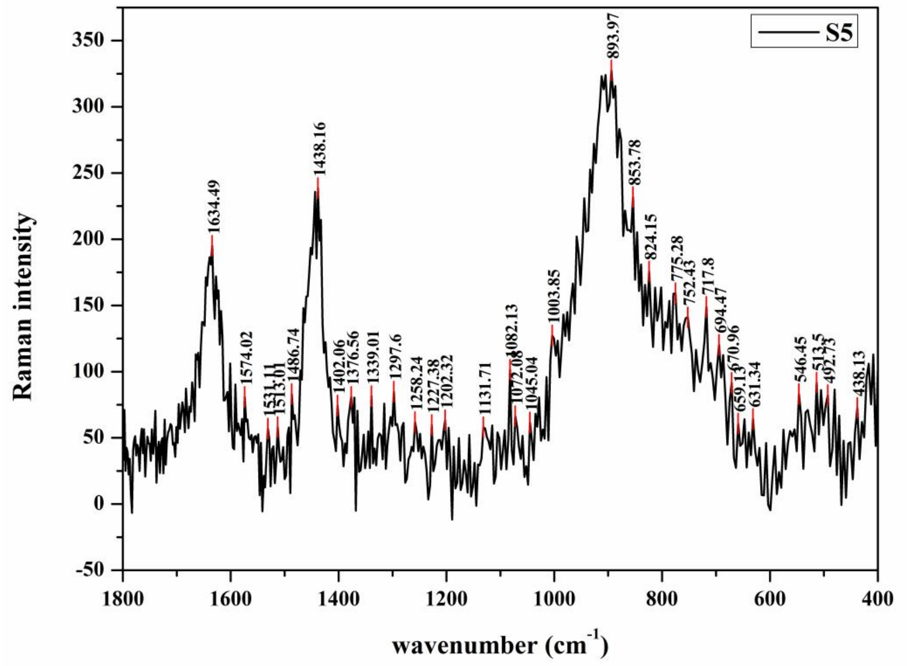

Figure S5. Raman spectrum of S5.

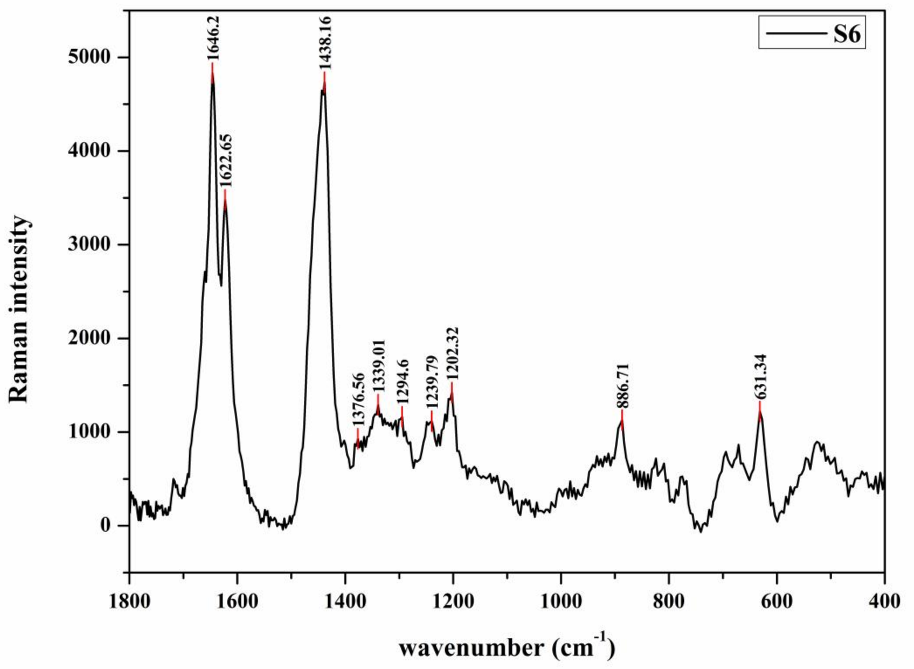

Figure S6. Raman spectrum of S6.

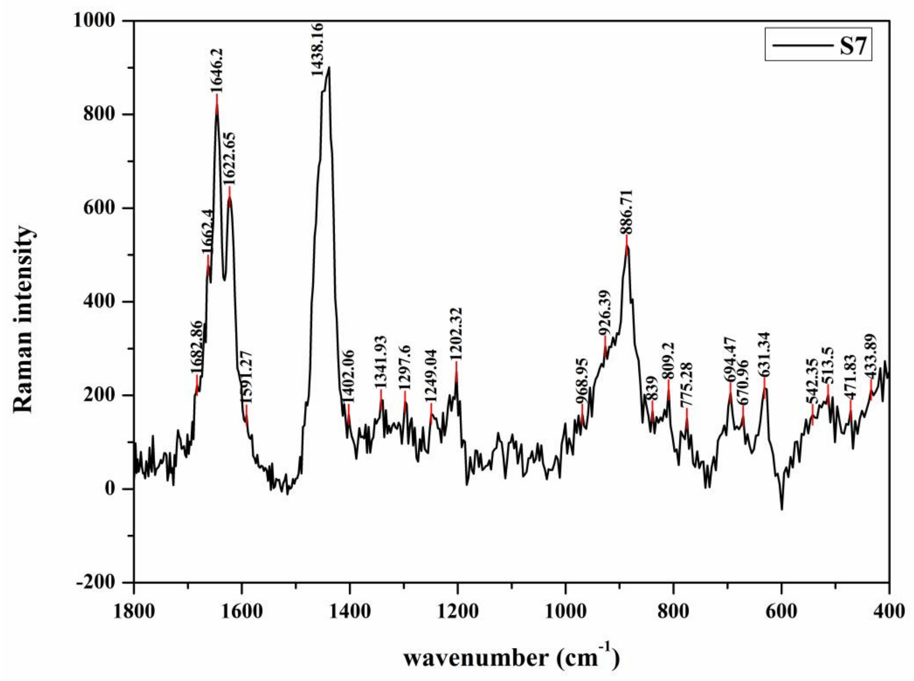

Figure S7. Raman spectrum of S7.

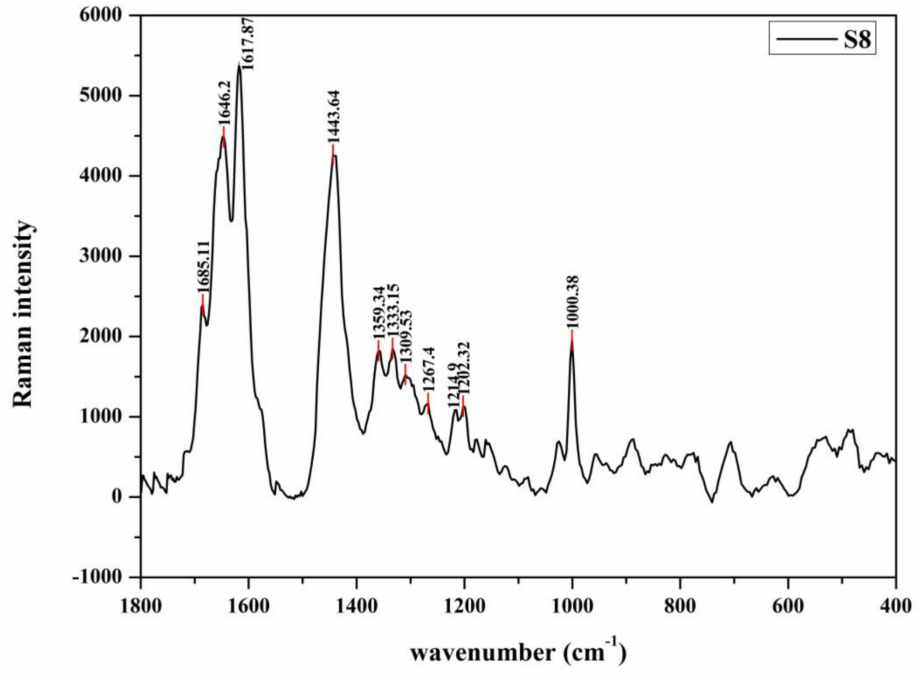

Figure S8. Raman spectrum of S8.

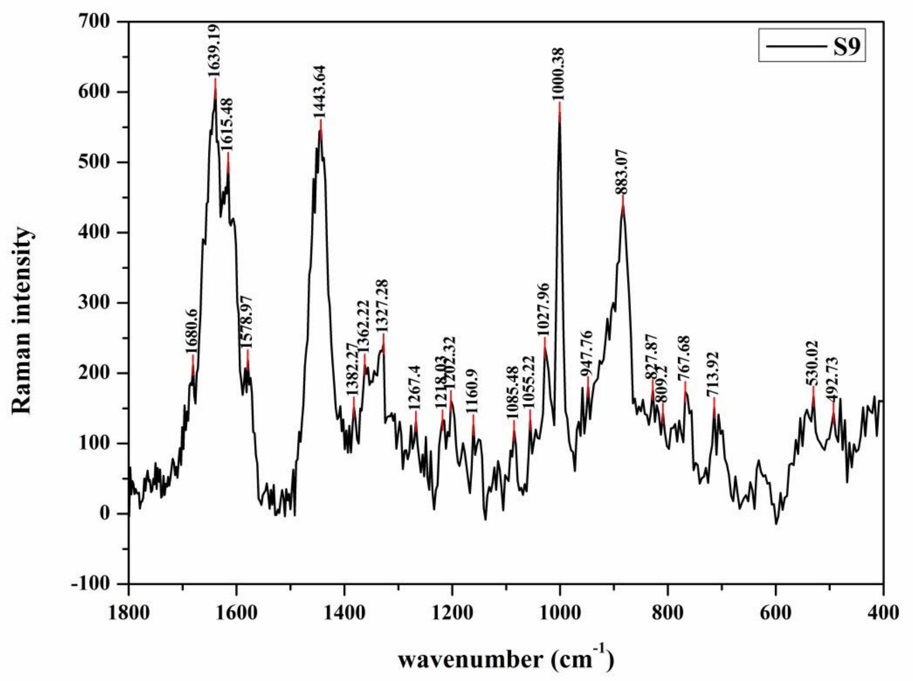

Figure S9. Raman spectrum of S9.

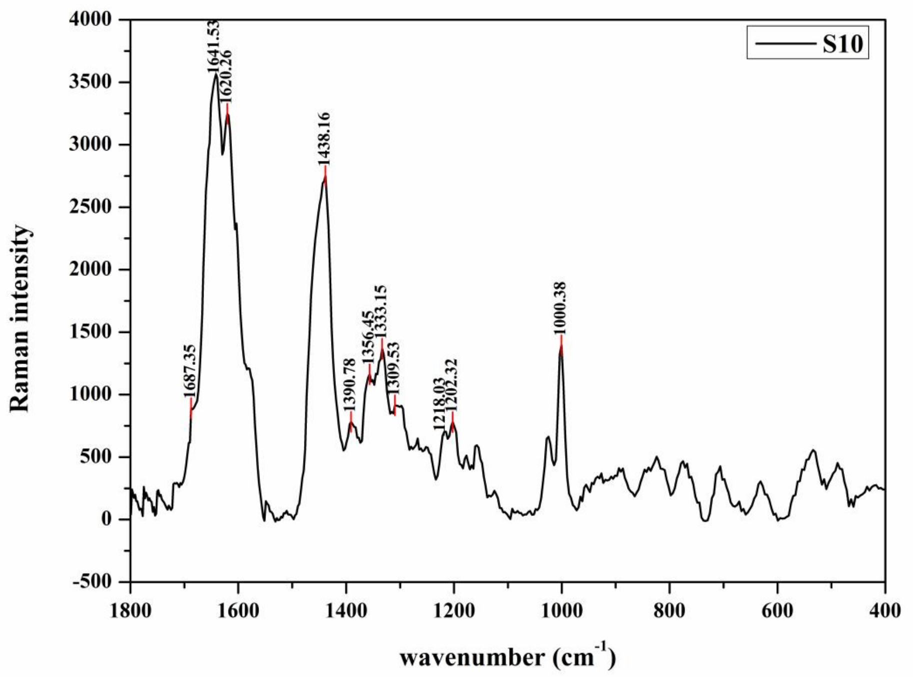

Figure S10. Raman spectrum of S10.

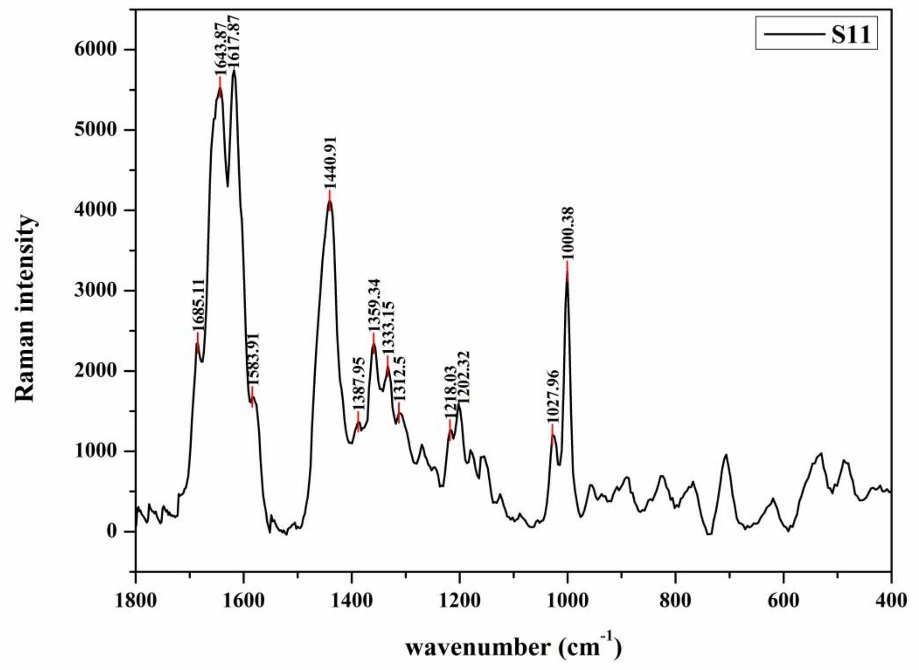

Figure S11. Raman spectrum of S11.

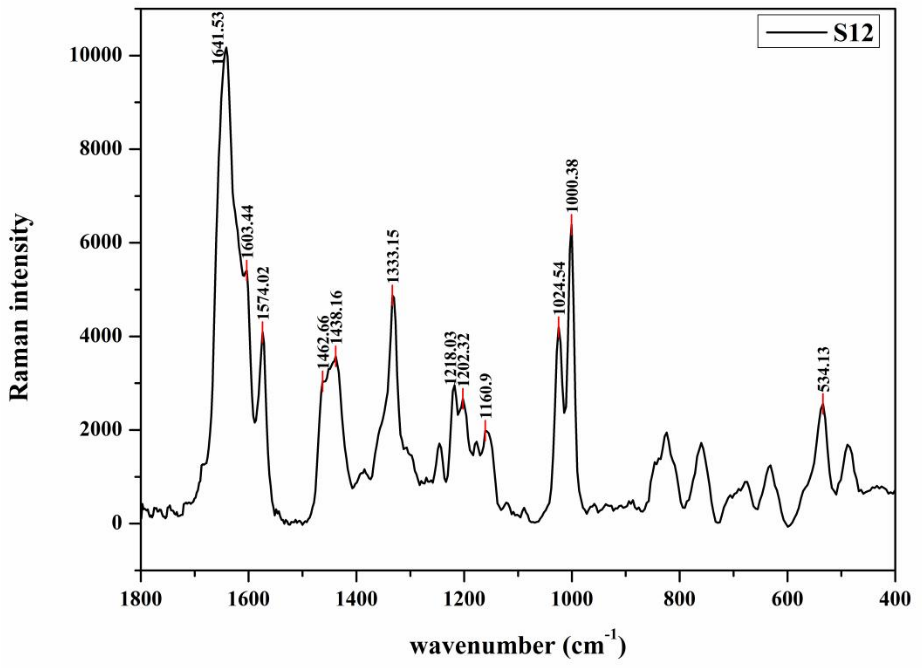

Figure S12. Raman spectrum of S12.

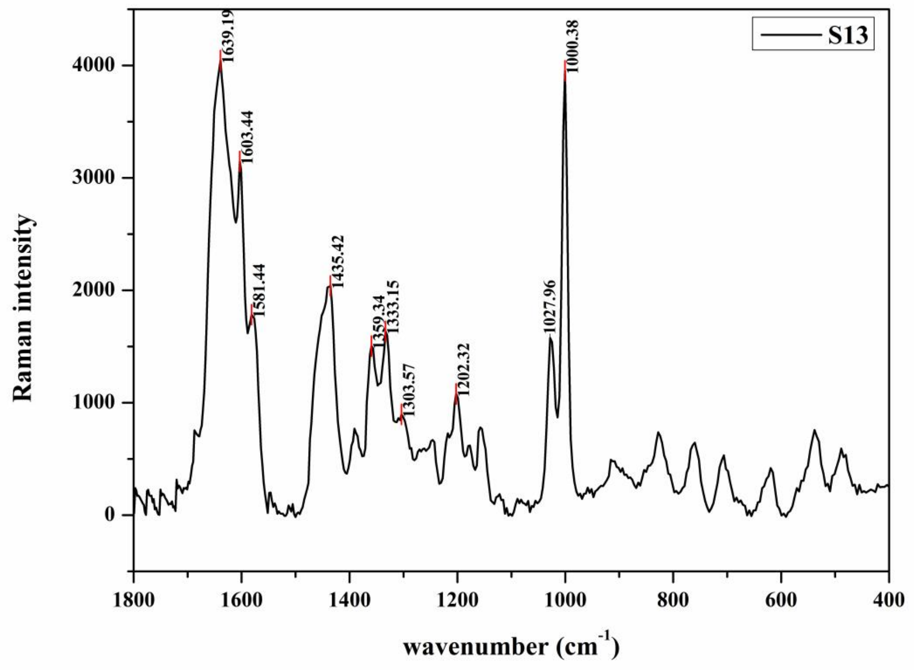

Figure S13. Raman spectrum of S13.

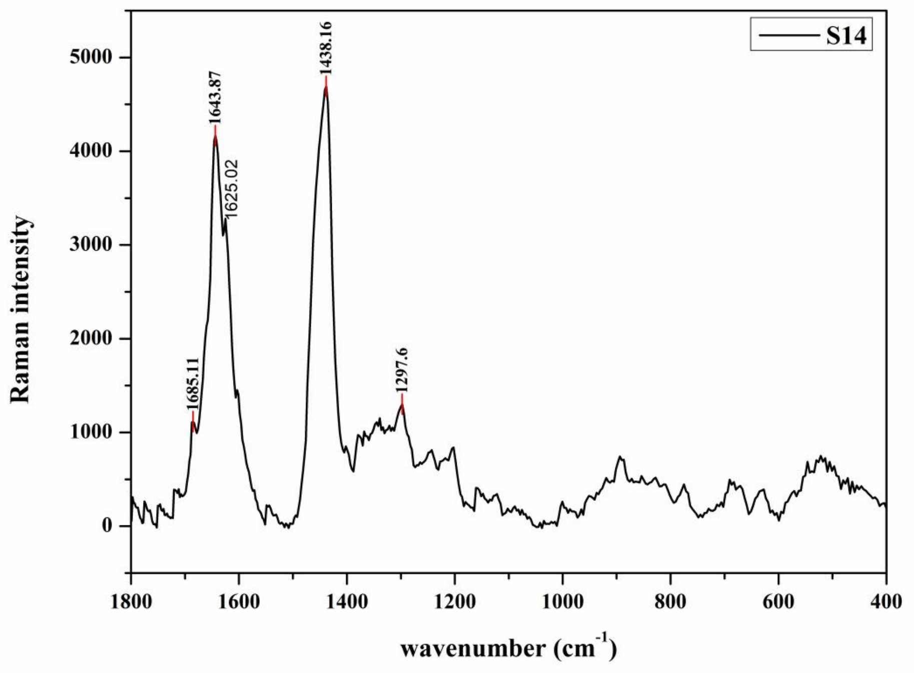

Figure S14. Raman spectrum of S14.

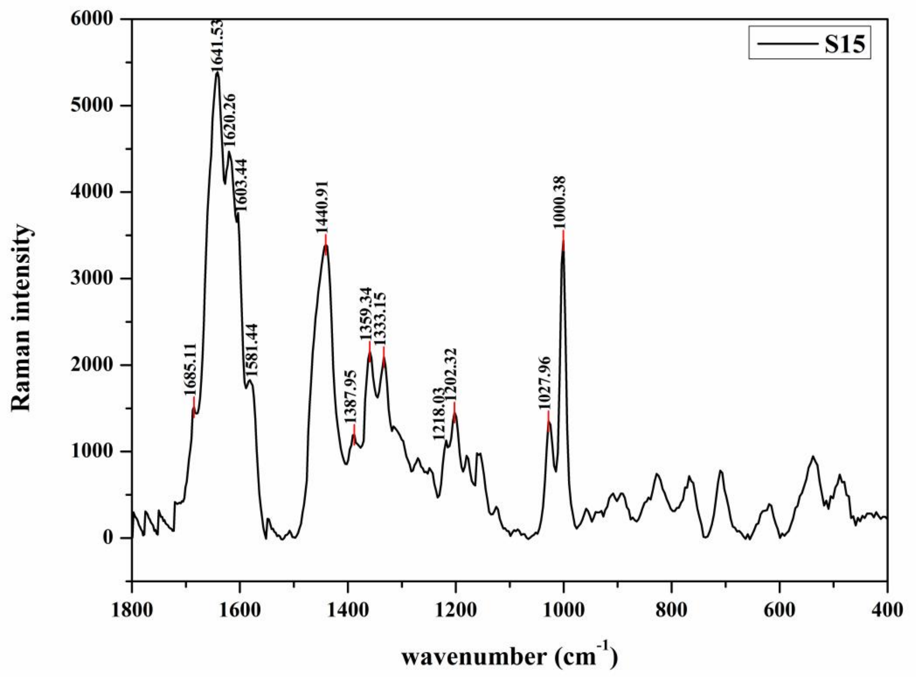

Figure S15. Raman spectrum of S15.

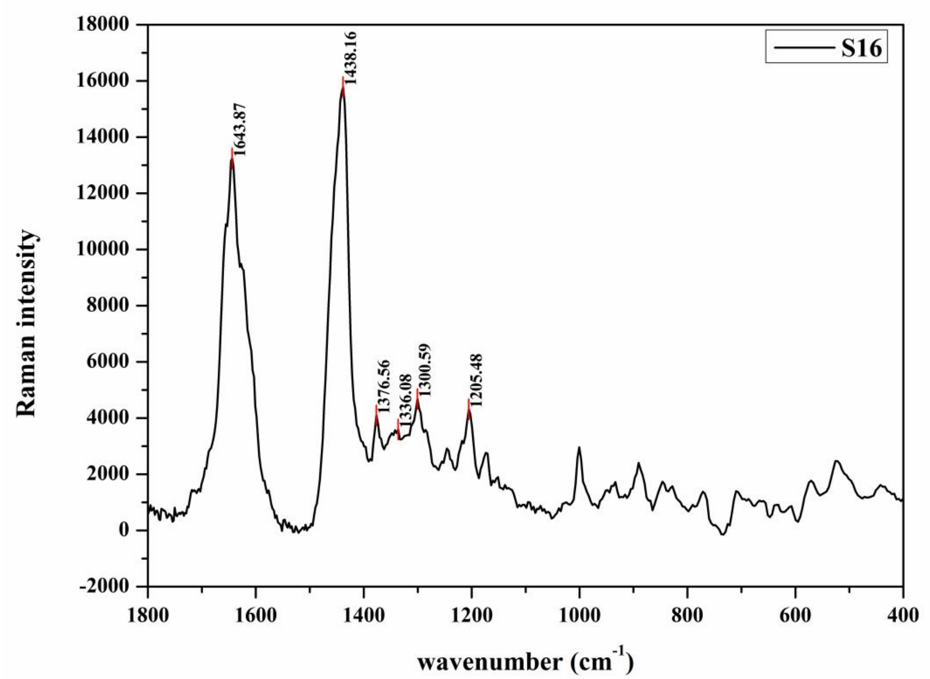

Figure S16. Raman spectrum of S16.

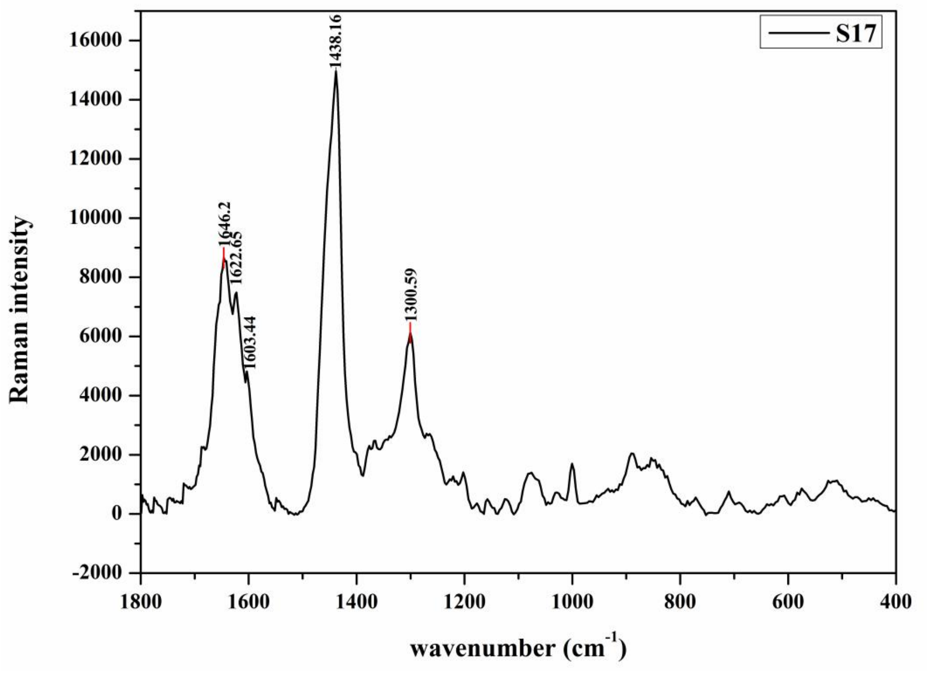

Figure S17. Raman spectrum of S17.

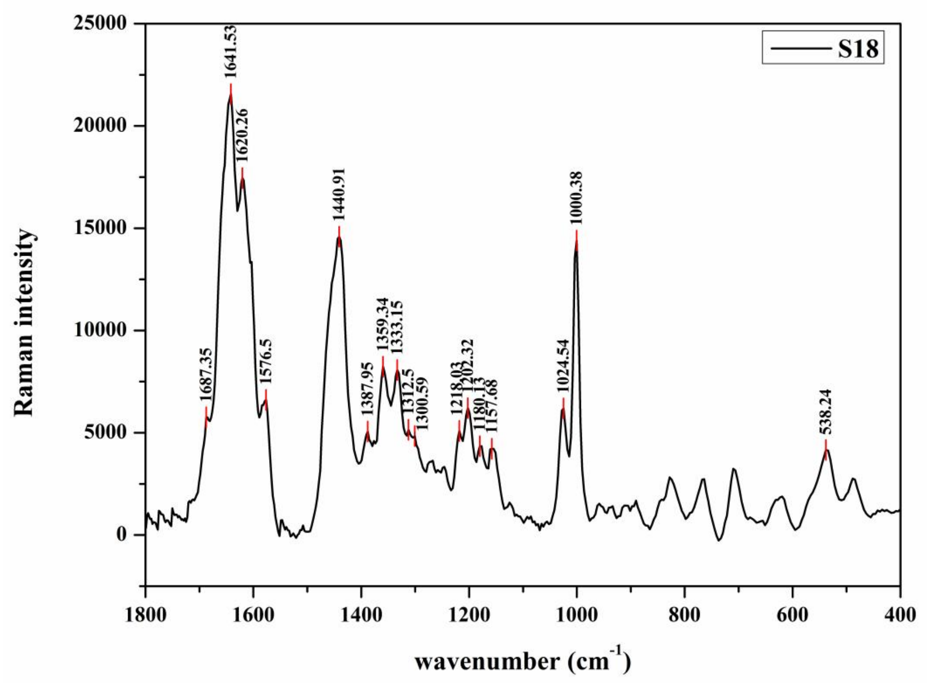

Figure S18. Raman spectrum of S18.

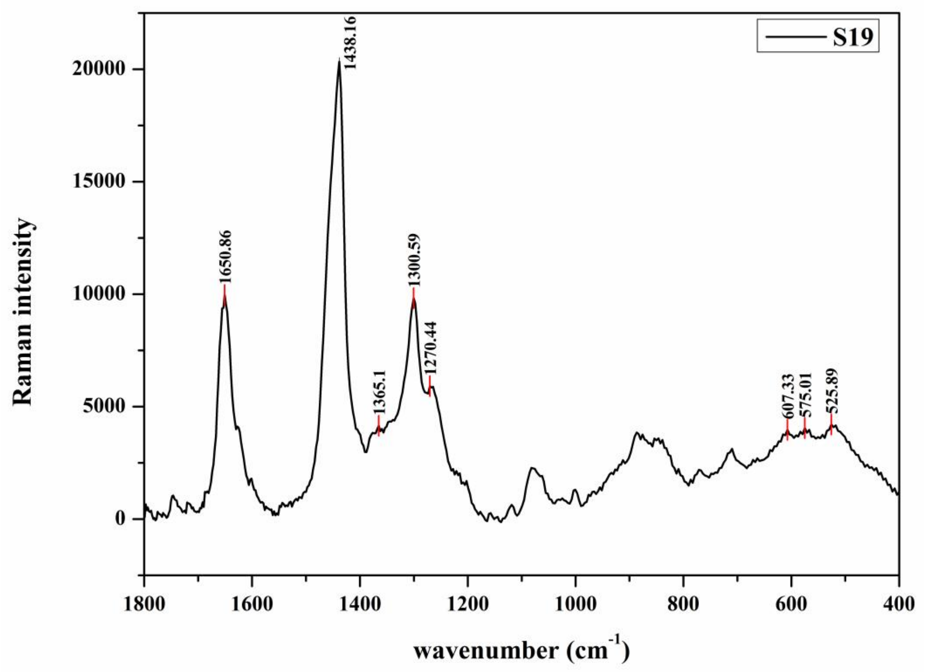

Figure S19. Raman spectrum of S19.

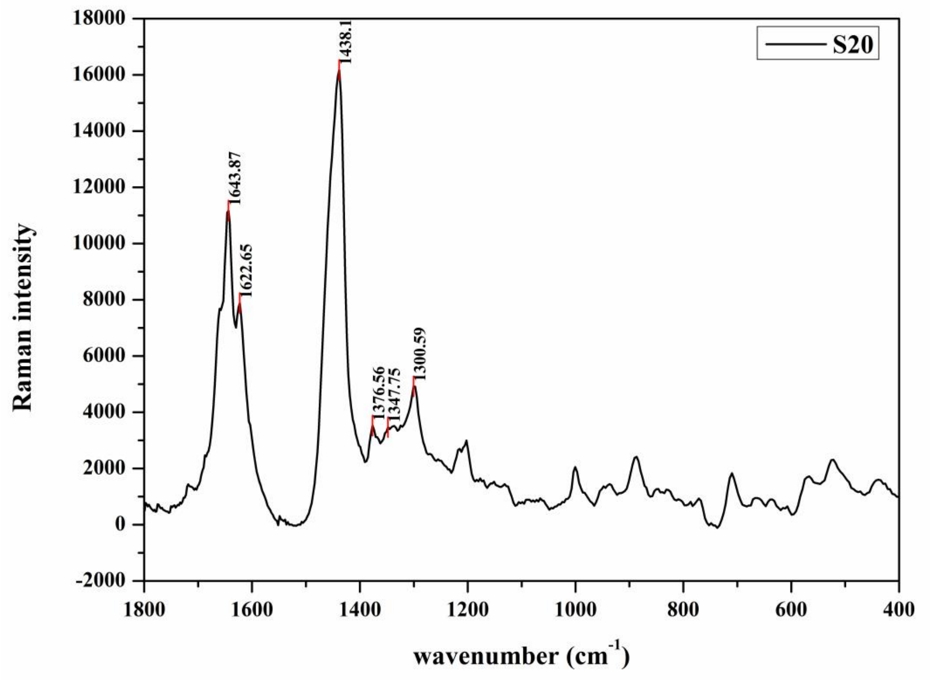

Figure S20. Raman spectrum of S20.

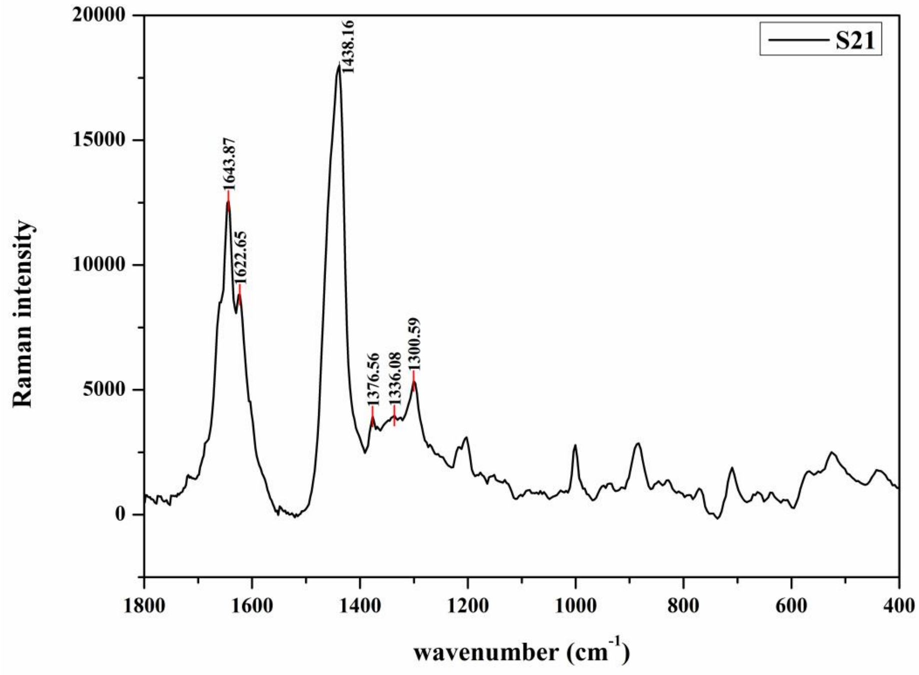

Figure S21. Raman spectrum of S21.

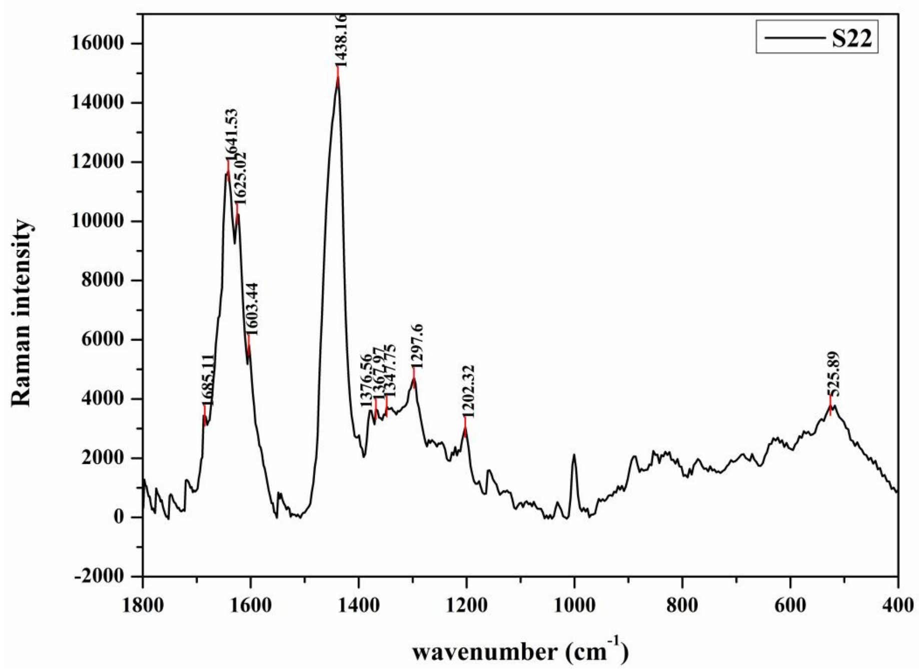

Figure S22. Raman spectrum of S22.

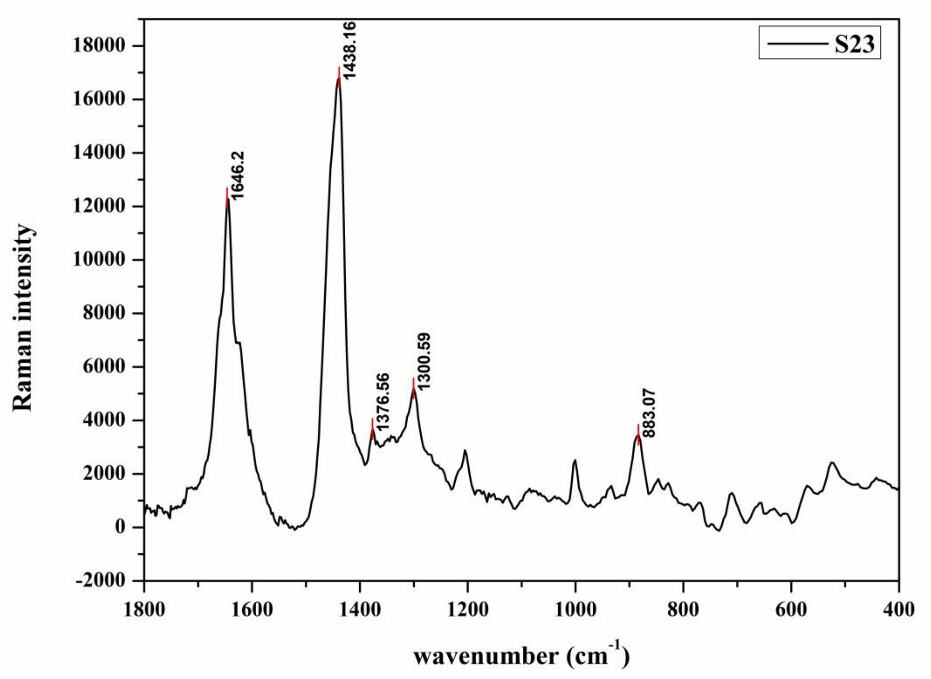

Figure S23. Raman spectrum of S23.

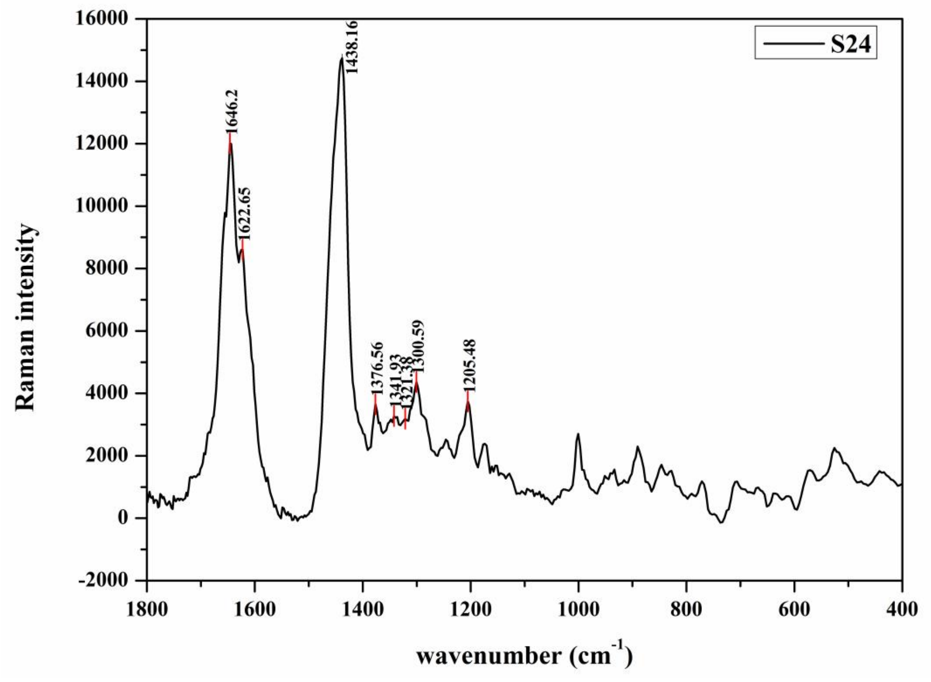

Figure S24. Raman spectrum of S24.

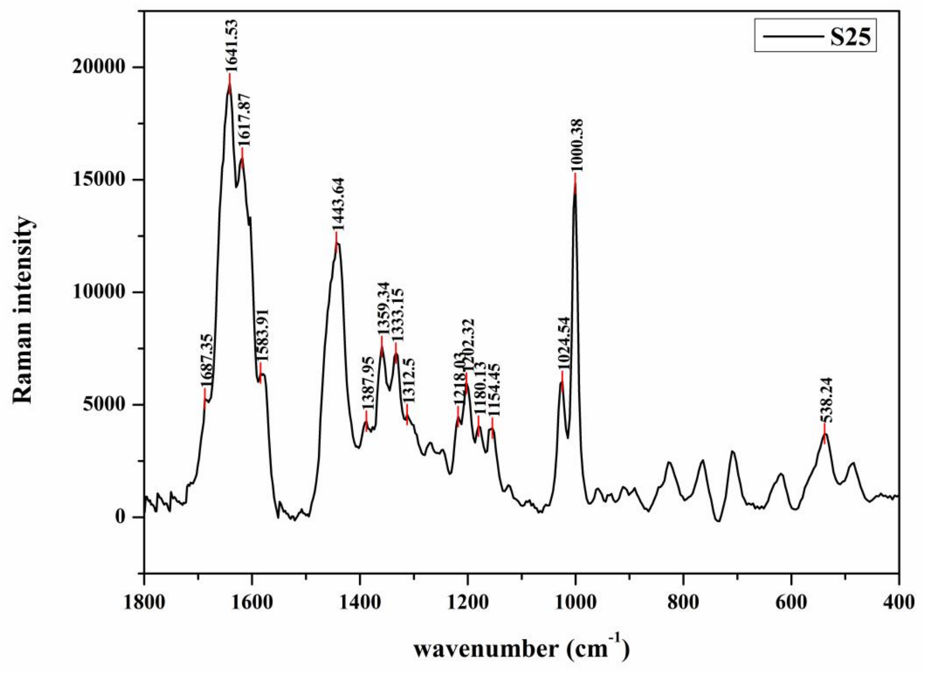

Figure S25. Raman spectrum of S25.

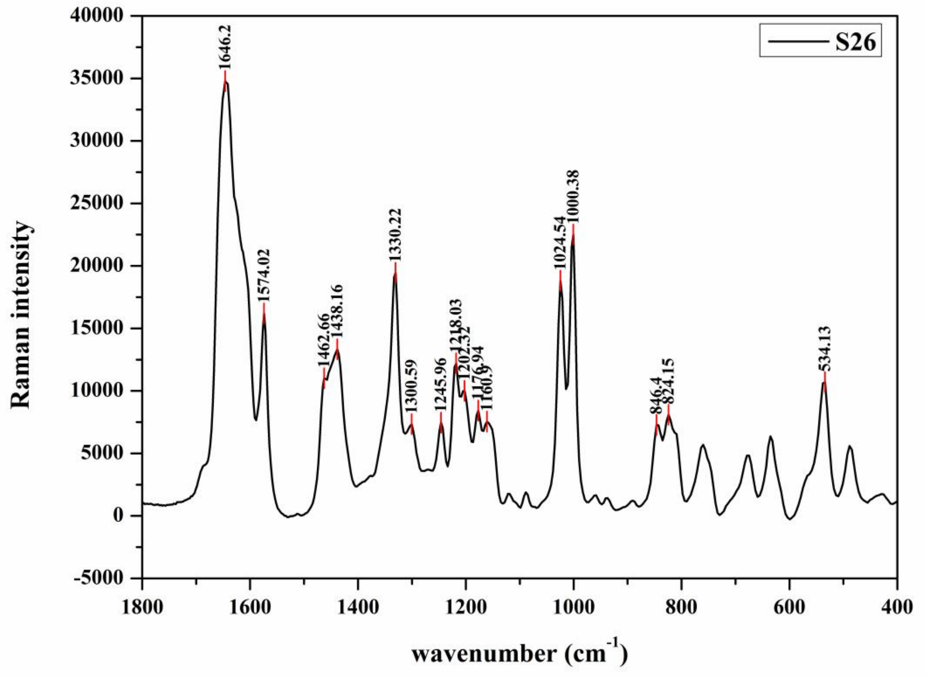

Figure S26. Raman spectrum of S26.

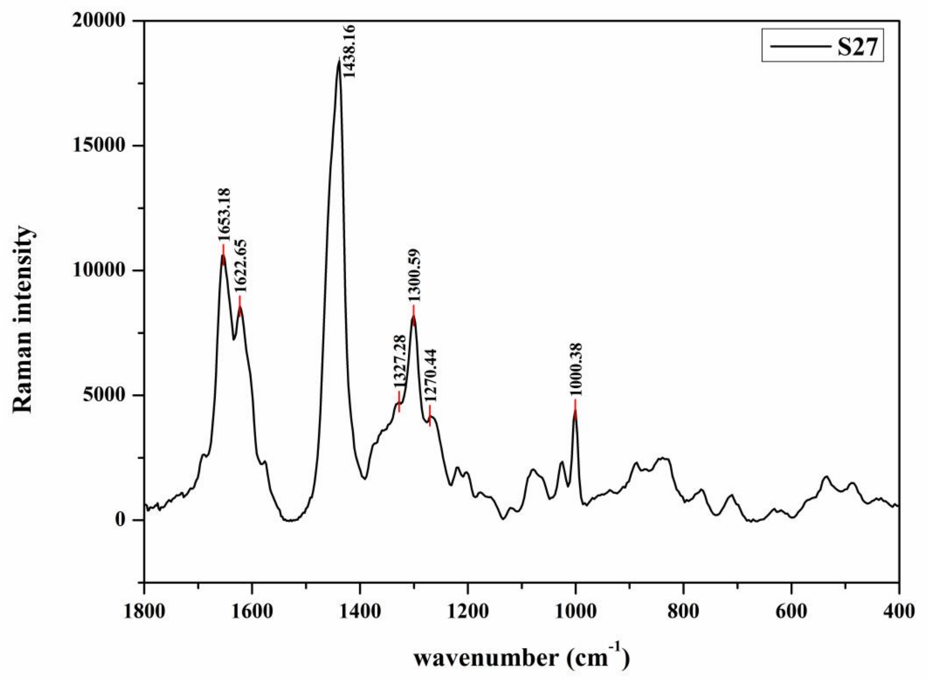

Figure S27. Raman spectrum of S27.

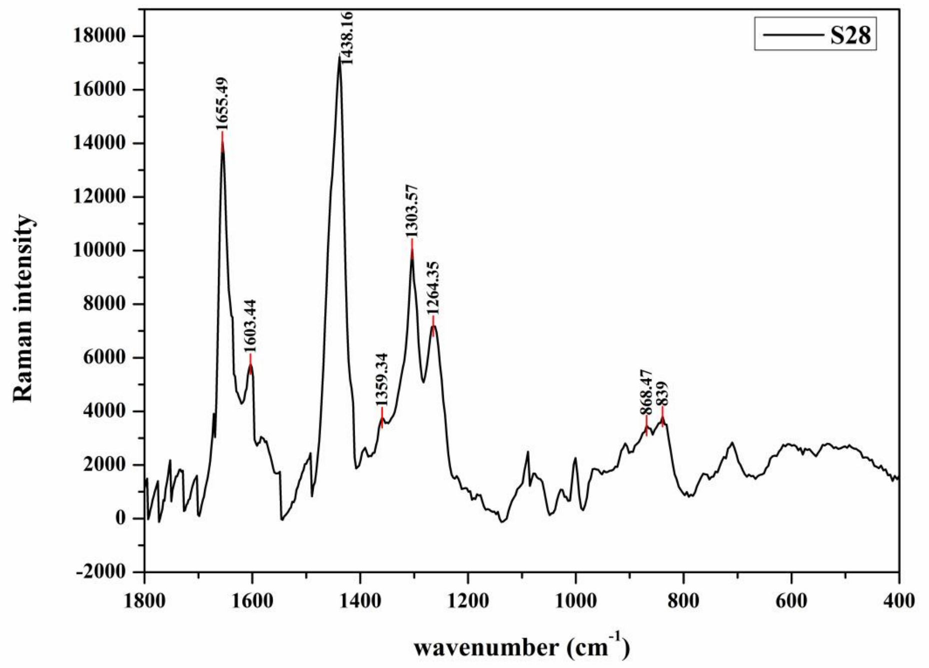

Figure S28. Raman spectrum of S28.

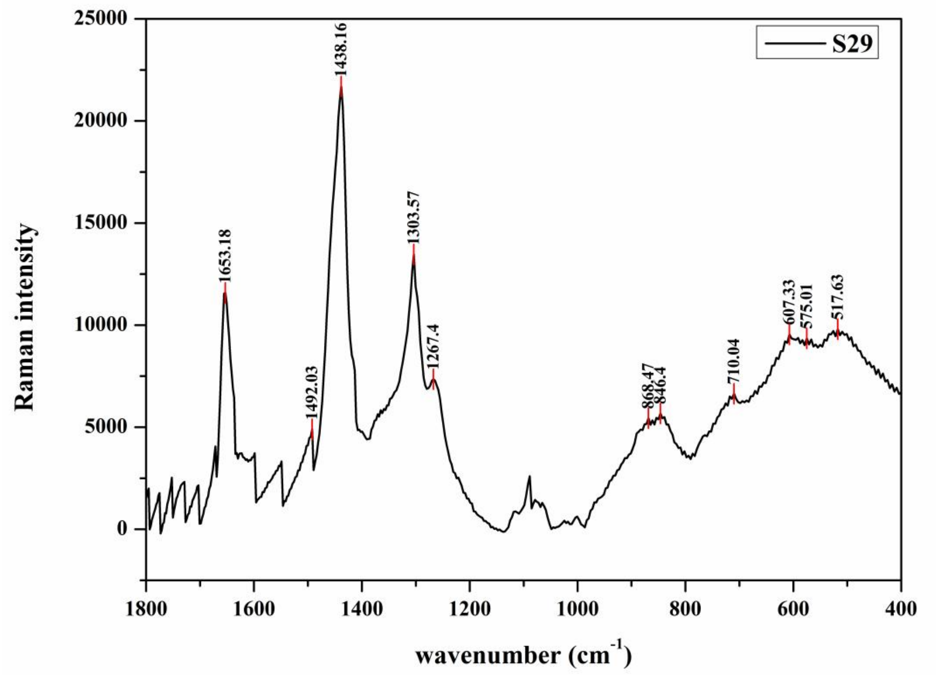

Figure S29. Raman spectrum of S29.

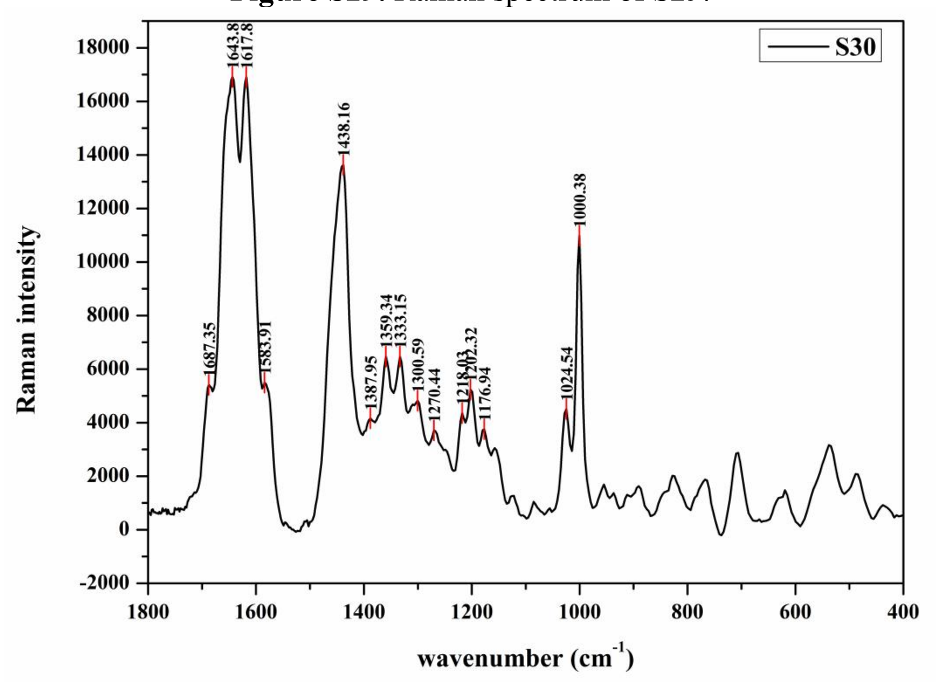

Figure S30. Raman spectrum of S30.

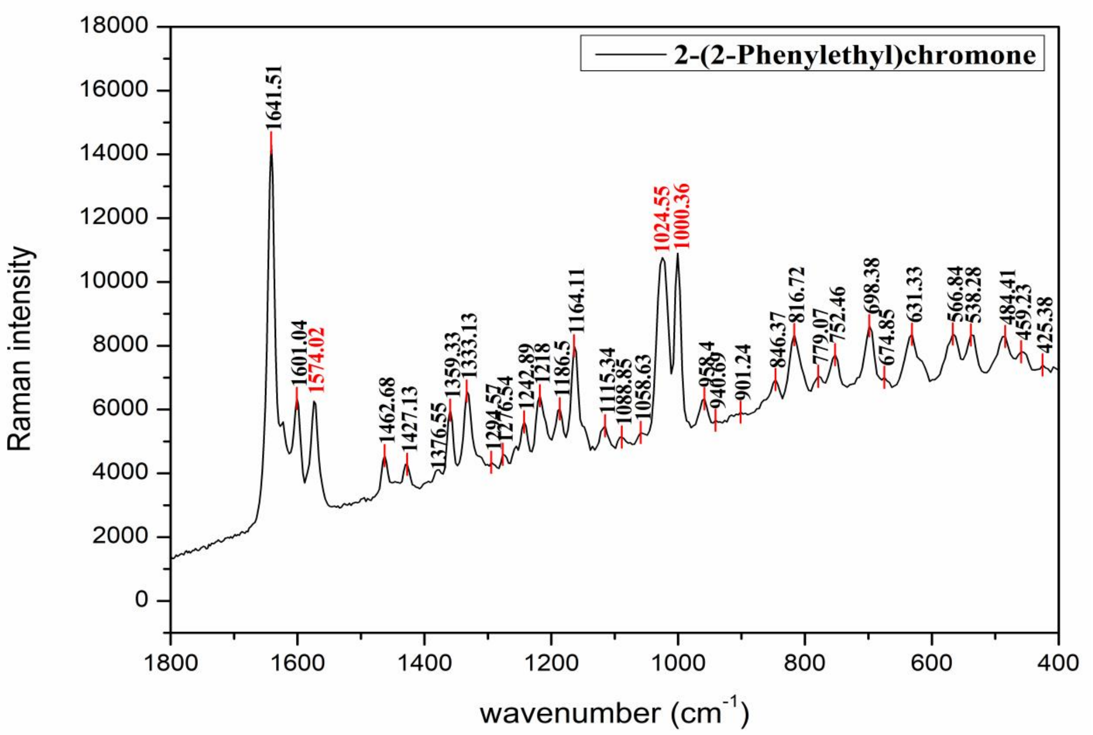

Figure S31. Raman spectra of reference material 2-(2-Phenylethyl) chromone.

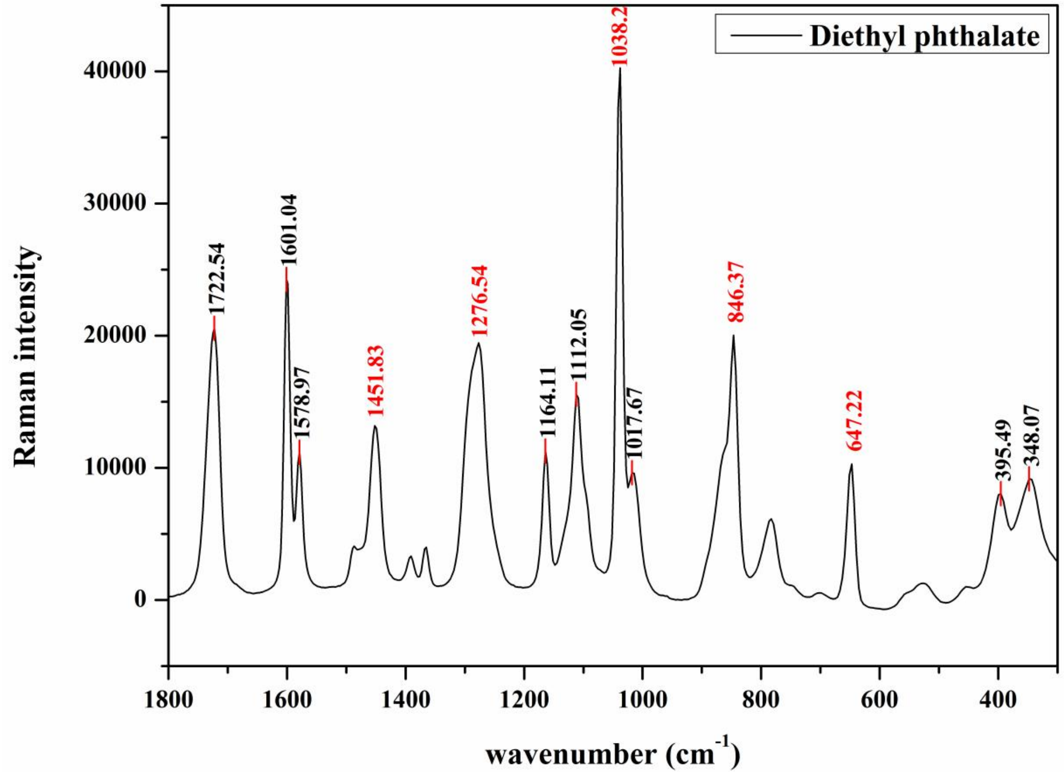

Figure S32. Raman spectra of reference material diethyl phthalate.

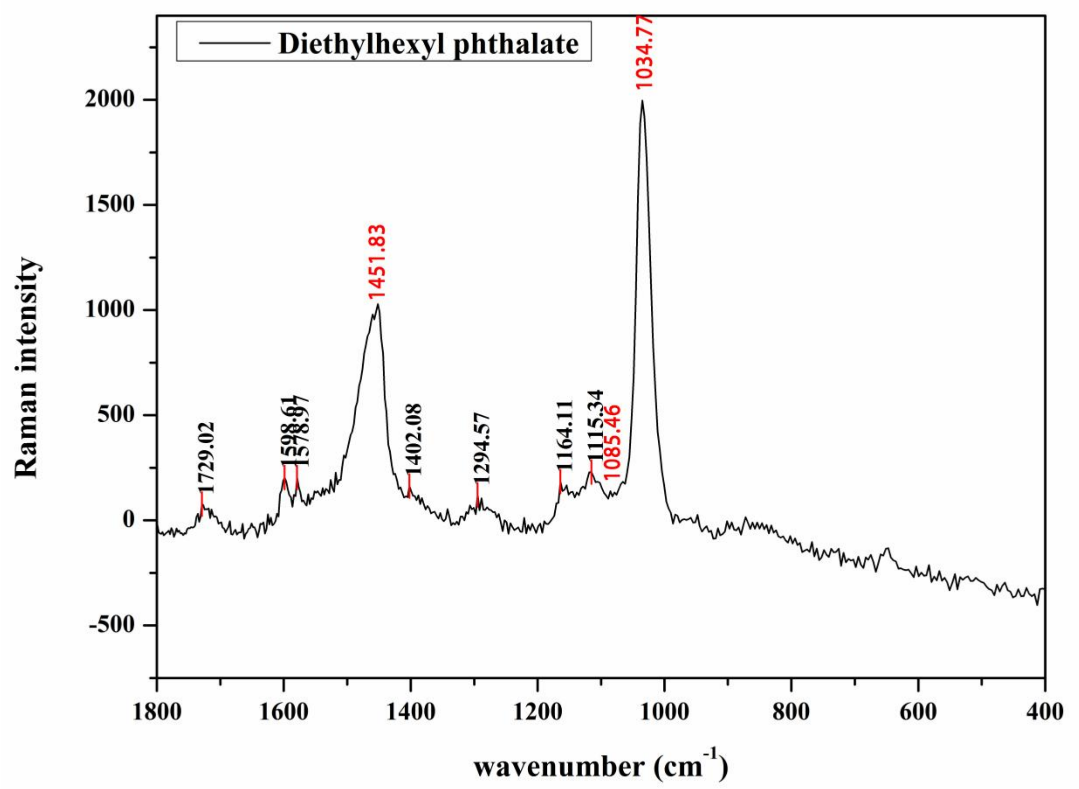

Figure S33. Raman spectra of reference material diethylhexyl phthalate.
